# Filaggrin as a potential biomarker in gastric cancer: insights from multi-omics analysis and experimental validation

**DOI:** 10.3389/fimmu.2026.1742982

**Published:** 2026-05-08

**Authors:** Nan Xia, Tianru Ben, Xin Guan, Linlin Gao, Yuan Yuan

**Affiliations:** 1 National Clinical Research Center for Medical Auxiliary Technology (Laboratory Medicine), Department of Laboratory Medicine, The First Hospital of China Medical University, Shenyang, Liaoning, China; 2 The First Clinical College of China Medical University, Shenyang, Liaoning, China; 3 Tumor Etiology and Screening Department of Cancer Institute and General Surgery, Key Laboratory of Cancer Etiology and Prevention, The First Hospital of China Medical University, China Medical University, Shenyang, China

**Keywords:** epithelial-mesenchymal transition, FLG, gastric cancer, multi-omics analysis, prognosis

## Abstract

**Background:**

Filaggrin (FLG) plays an important role in the progression of malignant tumors; however, its expression characteristics and biological functions in gastric cancer (GC) remain unclear.

**Methods:**

Cancer-related datasets were retrieved from public repositories, including the Gene Expression Omnibus (GEO) and The Cancer Genome Atlas (TCGA). A competing endogenous RNA (ceRNA) network was constructed to explore potential regulatory networks involving FLG. Differential expression analysis, genetic alteration analysis, and clinicopathological and survival analyses were performed to evaluate the role of FLG in GC. In addition, Gene Set Enrichment Analysis (GSEA), immune infiltration analysis, and in vitro functional experiments were conducted to investigate the biological effects and potential mechanisms of FLG in GC.

**Results:**

FLG was aberrantly expressed across multiple cancer types and was significantly associated with clinical characteristics and prognosis in GC. Further analyses showed that FLG was involved in genetic alterations and was closely associated with the immune microenvironment in GC. Functional experiments demonstrated that FLG promoted the invasion and metastasis of GC cells. Mechanistically, GSEA and experimental validation indicated that FLG exerted its tumor-promoting effects, at least in part, through activation of the epithelial-mesenchymal transition (EMT) signaling pathway.

**Conclusion:**

This study clarifies the biological role of FLG in GC and highlights its potential as a novel prognostic biomarker and therapeutic target. These findings provide new insights into the molecular mechanisms underlying GC progression and may contribute to the development of more effective diagnostic and therapeutic strategies.

## 1 Introduction

Cancer remains a leading cause of mortality worldwide, particularly in China and developed countries (1, 2). Despite advances in targeted therapies and immunotherapies, the clinical efficacy of current treatments remains limited for most solid malignancies, and the development of reliable biomarker-driven clinical trials continues to face significant challenges (2–5). Consequently, there is an urgent need to identify novel molecular biomarkers that can enhance our understanding of tumor development, improve prognostic stratification, and provide actionable therapeutic targets. Gastric cancer (GC), characterized by high incidence and mortality rates despite recent therapeutic progress (2), represents a paradigm of molecularly heterogeneous malignancy where multi-gene dysregulation drives oncogenesis. This complexity underscores the critical need for specific and reliable biomarkers to guide precision diagnosis and treatment strategies for GC.

Filaggrin (FLG) is a fundamental structural protein associated with epithelial cell differentiation and barrier integrity. Synthesized as a high-molecular-weight precursor, profilaggrin undergoes site-specific proteolytic processing by kallikrein-5 (KLK5) to generate functional filaggrin monomers (6). The FLG gene, located within the epidermal differentiation complex on chromosome 1q21.3, consists of three exons wherein exon 3 encodes the critical S100 calcium-binding domain, the B-domain, and 10–12 tandem filaggrin repeat sequences essential for keratin filament aggregation (7). Functional loss-of-function mutations in FLG constitute the primary genetic risk factor for atopic dermatitis and ichthyosis vulgaris (8, 9), establishing its indispensable role in maintaining epithelial homeostasis. Beyond dermatological disorders, emerging evidence indicates that FLG mutations and altered expression patterns are associated with various cancers, including breast, cervical, and colorectal malignancies (8, 10–15), suggesting a broader oncogenic involvement. Recent studies have further elucidated FLG’s roles in tumor progression and immune regulation (16, 17), establishing a foundation for investigating its specific functions in GC.

However, the role of FLG in GC remains particularly enigmatic and fundamentally distinct from its mutational impact in other tissues. While previous studies have characterized FLG mutations as structural drivers in breast cancer (18) or documented FLG loss during progression of cutaneous T-cell lymphoma (19, 20), the expression profile characteristics, prognostic implications, and mechanistic functions of wild-type FLG in GC tissues and cell lines remain virtually unexplored. Specifically, the relationship between FLG expression and critical clinicopathological feature, such as tumor staging, differentiation degree, and patient survival outcomes, has not been systematically analyzed, limiting the clinical translation of FLG as a potential biomarker for GC diagnosis and prognosis.

Moreover, the molecular regulatory networks through which FLG exerts its oncogenic effects in GC remain undefined. The competitive endogenous RNA (ceRNA) mechanism, wherein lncRNA-miRNA-mRNA interactions modulate gene expression, represents a critical layer of post-transcriptional regulation implicated in tumor proliferation, migration, and invasion. Simultaneously, immune cell infiltration patterns and immune checkpoint molecule expression within the tumor immune microenvironment (TIME) are increasingly recognized as determinants of GC progression and therapeutic response. Whether FLG functions through ceRNA networks to regulate malignant behavior, or how it modulates TIME and immune checkpoint pathways, remains entirely unexplored through multi-dimensional experimental approaches.

This study integrates comprehensive bioinformatics mining of public repositories (TCGA, GEO, HPA) with functional cellular experiments to systematically dissect the role of FLG in GC. By characterizing its expression patterns, prognostic value, genetic alterations, and immune regulatory associations, combined with mechanistic validation of EMT signaling through siRNA-mediated knockdown, we aim to establish FLG as a novel prognostic biomarker and potential therapeutic target, thereby providing a molecular foundation for precision medicine strategies in GC management.

## 2 Materials and methods

### 2.1 Research design and technical route

**Figure 1:**
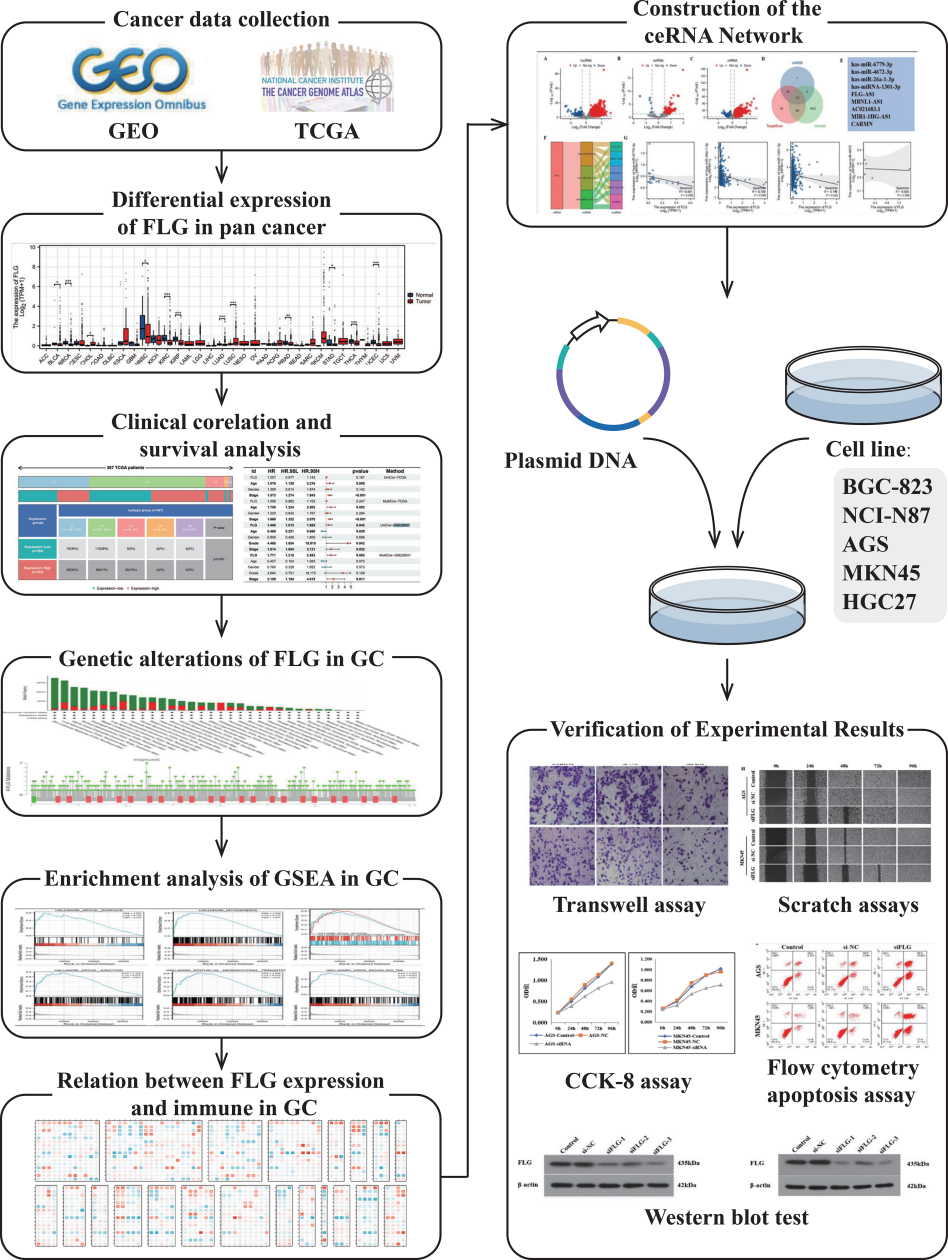
Research technical route flowchart: The technical route of this study is divided into (1) data collection and preprocessing; (2) bioinformatics analysis; and (3) experimental verification. Each step is interconnected, forming a closed-loop research from database mining to experimental verification, providing systematic evidence for analyzing the role of FLG in GC.

This study integrates bioinformatics analysis and cellular experimental validation to systematically dissect the mechanism of FLG in GC. The overall technical approach encompasses three major modules: data mining, bioinformatics analysis, and experimental validation. The specific process is illustrated in **Figure 1**.

### 2.2 Data acquisition and processing

Gene expression profiles and paired clinical information were downloaded from The Cancer Genome Atlas (https://portal.gdc.cancer.gov) to verify the expression and prognostic impact of FLG in GC based on the GPL570 platform tested by Affymetrix Human Genome U133 Plus 2.0 Array.

### 2.3 FLG expression analysis

We utilized Tumor Immune Estimation Resource version 2 (TIMER2) to analyze FLG expression levels between tumors and paired normal tissues. In tumor types with no or limited normal tissues, we investigated FLG expression by using ‘Gene Expression Profile’ section of Gene Expression Profiling Interactive Analysis (version 2) (GEPIA2) to combine data from normal tissues in the Genotype-Tissue Expression (GTEx) database, under parameters of log_2_ FC (fold change) cutoff = 1 and *P*-value cutoff = 0.01. Expression data were normalized using platform-specific algorithms: TCGA RNA-seq used TPM values; GEO (GSE66229) microarray used RMA normalization.

UALCAN tool was utilized to perform protein expression analysis. Protein expression data were from Clinical Proteomic Tumor Analysis Consortium (CPTAC) dataset. Besides, we downloaded and analyzed immunohistochemical images of FLG protein expression in human normal tissues and tumor tissues from the Human Protein Atlas (HPA).

### 2.4 Survival prognosis analysis and ROC curve analysis

We obtained overall survival (OS) and disease-free survival (DFS) significance maps and Kaplan–Meier (K-M) survival plots of FLG by utilized ‘Survival Analysis’ section of GEPIA2. We utilized R package p ROC (version 1.17.0.1) to analyze data and R package ggplot2 (version: 3.3.3) to visualize the results.

The PrognoScan database complies accessible tumor microarray datasets, facilitating the correlation of gene expression and survival including OS and disease-free survival (DFS). We evaluated the relationship between FLG expression and prognosis in diverse tumor types using the PrognoScan database (http://www.abren.net/PrognoScan/) with a threshold *P*-value of 0.05.

For meta-analysis of FLG prognostic value, inclusion criteria were: studies reporting FLG expression with extractable survival data (HR or Kaplan-Meier curves) in GC patients; exclusion criteria: reviews, case reports, and studies lacking survival outcomes. Random effects model was selected due to significant heterogeneity between cohorts (*I²* > 50%, *P* < 0.10).

### 2.5 Genetic alteration analysis

The cBioPortal tool and GSCALite were employed to analyze FLG genetic alterations (21). Alteration frequency and type and mutated site for FLG from TCGA were obtained and analyzed. We input ‘FLG’ to ‘Quick Search’ module, and genetic alterations and mutated site data can be obtained from ‘Cancer Types Summary’ and ‘Mutations’ modules.

High-frequency mutations were defined as≥2% alteration frequency in the TCGA-STAD cohort (cBioPortal). For pathway enrichment post-mutation, we applied GSEA criteria: |NES|>1.4 and FDR<0.25, selecting pathways (E2F targets, EMT) relevant to FLG’s biological functions in epithelial cell regulation.

### 2.6 Immune infiltration analysis

MCPCOUNTER, TIDE and EPIC algorithms were employed to investigate the correlation between FLG expression and infiltration of CAFs in the ‘Immune Association’ section of TIMER2.

We obtained the tumor dataset from the UCSC database. Immediately, the FLG expression data in each sample were extracted. Besides, tumor gene expression profiles were extracted. Subsequently, we mapped them to GeneSymbol. Finally, ESTIMATE was used to calculate stromal, immune, and estimate scores, and the corr. test was utilized to calculate Pearson’s correlation between FLG expression and immune infiltration scores.

TIMER2 (MCPCOUNTER/TIDE/EPIC) used default settings with Spearman correlation, significance *P* < 0.05; ESTIMATE used default stromal/immune gene signatures; correlation analysis used Pearson R>0.2, *P* < 0.05.

### 2.7 Construction of network and FLG-related gene enrichment analysis

TargetScan, miRDB, and miraids databases were used to predict the FLG ceRNA network. GeneMANIA website was utilized to find functionally similar genes using a large body of genomics and proteomics data, and we used it to search for genes that are functionally similar to FLG. Also, a human FLG-binding protein co-expression network can be obtained by STRING tool. All gene symbols were used as input gene symbols for Gene Ontology pathway enrichment analysis. (|log_2_fold change|≥1.0, adjusted *P* < 0.05; correlation |R|≥0.4, *P* < 0.05.

### 2.8 Cell culture

The human GC cell lines BGC-823, NCI-N87, AGS, MKN45, and HGC27 were maintained in our laboratory, and GES-1 cells were generated at the Beijing Cancer Hospital. Cells were cultured in an incubator containing 5% fetal bovine serum at 37°C with 5% CO_2_.

### 2.9 Plasmids construction and cell transfection

Three independent siRNA sequences targeting distinct, non-overlapping regions of FLG mRNA (all synthesized by JiKai) were transfected into AGS and MKN45 cells using Lipofectamine 3000 (Invitrogen). Preliminary validation confirmed that all three siRNAs significantly reduced FLG expression, with siFLG-3 exhibiting the highest knockdown efficiency (>80% at protein level) and being selected for comprehensive phenotypic characterization.

### 2.10 RNA extraction and qRT-PCR

Total RNA was extracted with TRIzol, and cDNA was generated using a reverse transcription kit. The mRNA products and specific primers were used for qRT-PCR. β-actin was used as an endogenous control. All experiments were repeated thrice.

### 2.11 Western blotting

After quantification and denaturation, protein was subjected to 10% SDS-PAGE and transferred to a polyvinylidene difluoride membrane. The membrane was incubated separately with primary antibodies against anti-human FLG antibody (1:500 dilution), against anti-human N-cadherin antibody (1:1000 dilution), against anti-human E-cadherin antibody (1:1000 dilution), against anti-human Vimentin antibody (1:1000 dilution), against anti-human ZEB1 antibody (1:500 dilution) and a mouse anti-human *β*-actin (1:500 dilution). The secondary antibody, horseradish peroxidase (HRP)-conjugated goat anti-rabbit or anti-mouse antibody was used as a 1:5000 dilution. The film is scanned and the optical density value of the target strip is analyzed with the Gel image processing system.

### 2.12 CCK8 assay

GC cells transfected with si-FLG and si-NC in logarithmic growth phase were seed in 96-well plates at 5×10^3^ cells/well density, and 5 multiple wells were designed for each group. After incubation for 0h, 24h, 48h, 72h and 96h, the supernatant was discarded and 100μl complete medium was added to each well, then a volume of 10μl CCK8 solution was added to each well and incubated in an incubator for 1h. The wavelength at OD450 was measured in a multiplate reader.

### 2.13 Flow cytometry assay

GC cells transfected with si-FLG and si-NC in logarithmic growth phase were cultured in 6-well plates with 5×10^5^ cells/well. The cells of each group were centrifuged at 150g for 5min and then collected, the supernatant was carefully removed. The cells were washed with PBS twice and resuspend with 500μl binding Buffer. According to manufacturer’s instructions, samples were stained with Annexin V-FITC and propidium iodide. Then, incubation at room temperature for 15min away from light and an Aria flow cytometer was utilized to display the apoptotic cell population. Finally, FlowJo software was used to analyze data.

### 2.14 Transwell assay

Single cell suspensions (2.5×10^4^ cells/well) were added to the upper chambers and allowed to invade for 24 h at 37 °C in a CO_2_ incubator. Cells remaining attached to the upper surface of the filters were carefully removed with cotton swabs. The membranes were fixed with 4% paraformaldehyde for 20 min, and stained with 0.1% crystal violet for 20 min at room temperature and examined by light microscopy.

Cell invasion was assayed in BD Bio-coat Matrigel invasion chambers with an 8-μm pore size polycarbonate filter coated with Matrigel. DMEM (500μl) containing 10% FBS was added to the lower chambers. Data are presented as mean ± SD of three independent experiments.

### 2.15 Wound healing assay

According to the experimental plan, cells of each group were cultured until the density was fused. Before the experiment, the medium was changed to serum-free medium and 10μg/ml of mitomycin C was added. Cells in each group were scratched with 200ul pipette tip, and the surface was cleaned with serum-free medium once to remove the cell debris, and then cultured with serum-free medium. Cells were evaluated at 0, 24, 48, 72 and 96h using an inverted phase-contrast microscope.

### 2.16 Statistical analysis

Results from the Oncomine database included fold change, p-value, and gene rank. The PrognoScan and GEPIA databases produced HR and *P*-values or Cox *P*-values according to a log rank test. The correlation between FLG expression and other related immune genes was investigated using the Spearman method and the correlation strength was categorized according to R values. R values of 0.80-1.00 were deemed very strong, R values of 0.60-0.79 were strong, R values of 0.40-0.59 were moderate, R values of 0.20-0.39 were weak, and R values of 0.00- 0.19 were categorized as very weak.

Data were analyzed using GraphPad Prism (version 6.00) and SPSS (version 20.0). We employed One-way ANOVA to compare multiple groups, and Student’s t-test to compare two groups. Data were reported as means ± SD. *P* < 0.05 was considered statistically significant.

## 3 Results

### 3.1 Identification of FLG expression levels and protein–protein interactions in GC

**Figure 2:**
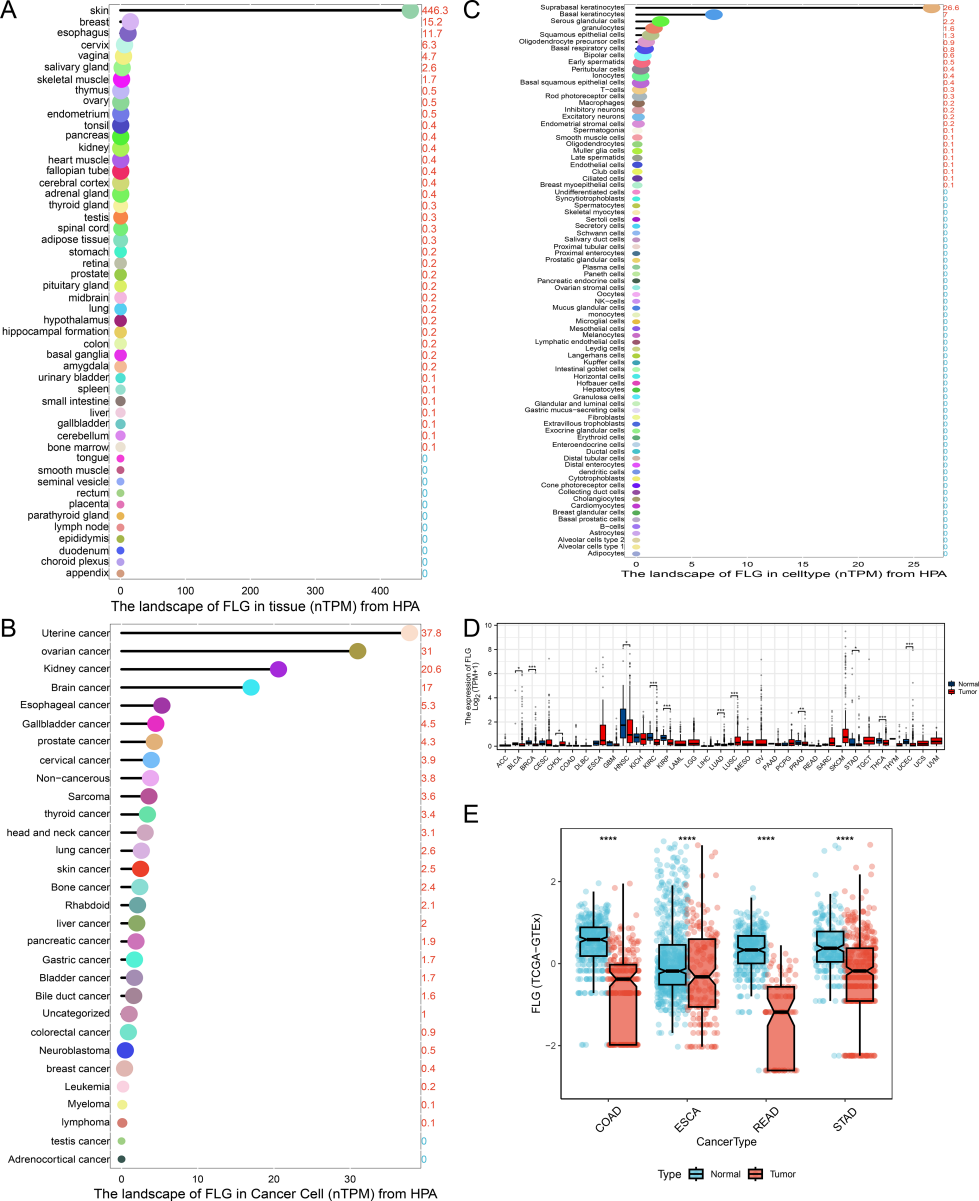
Differential expression of FLG in pan cancer. Based on the HPA database, differential expression of FLG in normal tissues/organs **(A)** and tumor cells **(B)**. Cell type analysis was displayed **(C)**. TCGA database to detect FLG expression in different malignant tumors **(D)**. TCGA and GTEx we’re analyzed FLG expression in COAD, ESCA, READ and STAD compared to paired normal tissues **(E)**.

First, based on the HPA database, we found significant differences in the expression of FLG in normal tissues and organs, exhibiting the highest expression level in the skin, and almost zero expression in the lymph nodes, epididymis, and other regions (**Figure 2A**). Furthermore, it exhibits high expression in tumor cells such as uterine cancer, ovarian cancer, and kidney cancer, whereas its expression approaches zero in adrenal cortex cancer and testis cancer cells (**Figure 2B**). Subsequent to this, cell type analysis revealed that FLG displayed higher expression levels in suprabasal keratinocytes, basal keratinocytes, serous glandular cells and granulocytes, whereas its expression was nearly undetectable in various cell types such as adipocytes and B cells (**Figure 2C**). Next, we used TCGA database to detect FLG expression in different malignant tumors. FLG levels were downregulated in STAD and other 10 tumor types. In contrast, FLG expression was upregulated in CHOL and LUSC (**Figure 2D**). Finally, we used TCGA and GTEx to analyze FLG expression and found that FLG expression was downregulated in COAD, ESCA, READ and STAD compared to paired normal tissues (**Figure 2E**). Starting from “normal tissues, cancer cell lines, cell types, pan-cancer, and specific cancer types”, we first depict the basic expression profile of FLG through the HPA database. Then, by combining cancer databases, we narrow down the scope to tumor tissues/cells, and ultimately focus on specific cancer types for comparison. This provides preliminary evidence of “abnormal expression” for subsequent research on the functional mechanisms of FLG in GC.

**Figure 3:**
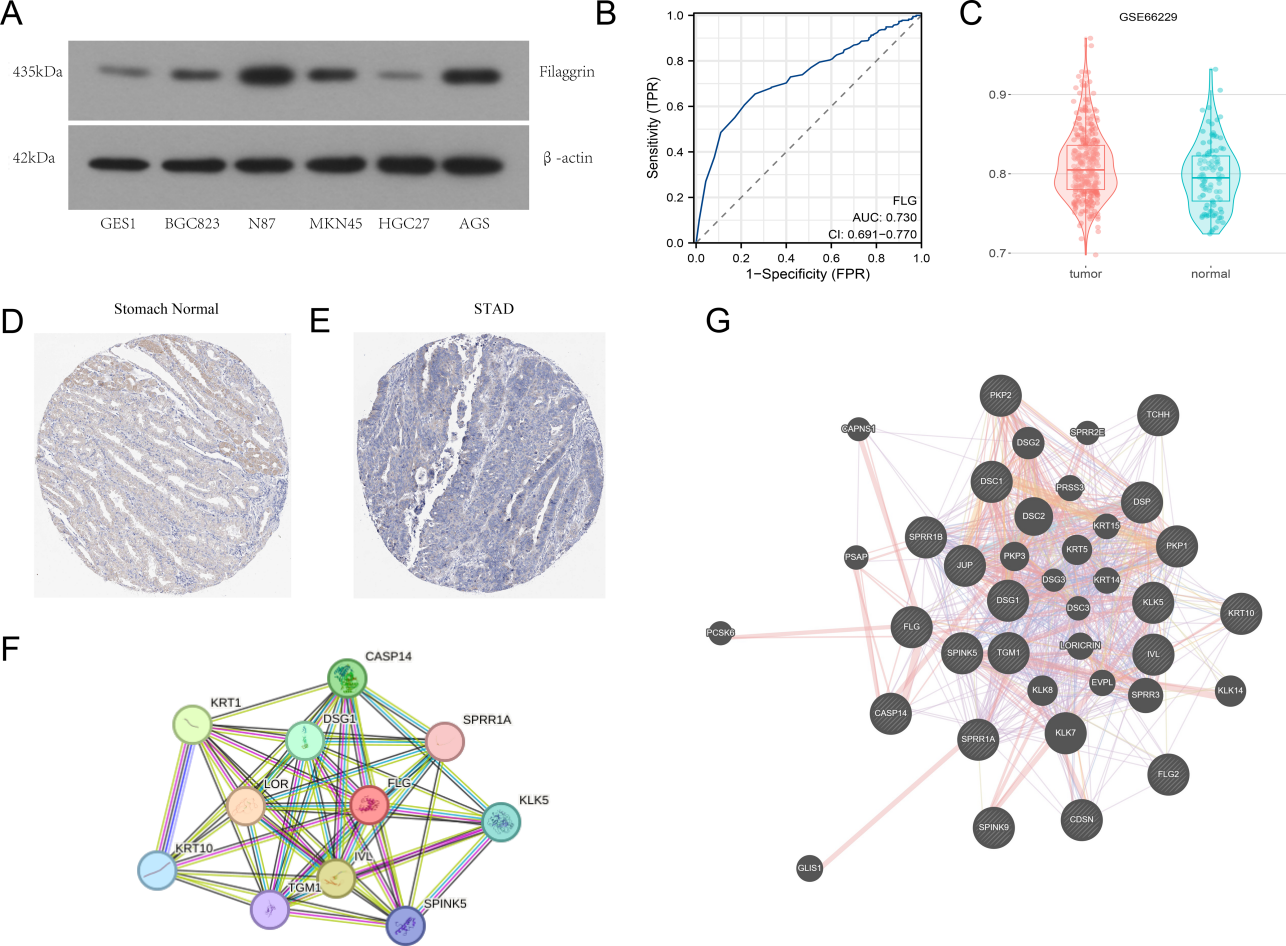
Identification of FLG expression levels and protein–protein interactions in GC. **(A)** Western Blot assay was conducted to verify the expression level of FLG protein in GC cells. **(B)** ROC curve was used to validate the diagnostic value of FLG in patients with GC. **(C)** Box plot was employed to show the expression difference of FLG in GC across different TCGA datasets. **(D, E)** Expression and localization of FLG protein in GC tissues and paired normal gastric tissues from the HPA database (D represents normal gastric tissue, E represents GC tissue; brown color indicates positive FLG staining). **(F, G)** FLG interaction networks were constructed using databases such as STRING (F stands for the core interaction network, and G stands for the extended interaction network).

To investigate the expression level of FLG protein in GC cells, we selected GES1 and GC cells (BGC823, N87, MKN45, HGC27, and AGS). Western blotting revealed that the FLG expression was higher in GC cell lines than in GES1 (**Figure 3A**). Quantitative Western blotting analysis revealed that FLG protein expression was significantly upregulated in GC cell lines compared to GES-1. Densitometric quantification of band intensities (normalized to *β*-actin) from three independent experiments showed that FLG levels were markedly elevated in NCI-N87 (3.2 ± 0.5 fold), AGS (2.9 ± 0.4 fold), MKN45 (2.3 ± 0.6 fold) and BGC-823 (2.0 ± 0.3 fold) cells relative to GES-1 (*P* < 0.01 for all comparisons, one-way ANOVA). The AUC for FLG was 0.730, suggesting that FLG possesses significant diagnostic value for GC (**Figure 3B****).** The GEO dataset, GSE66229, indicated that FLG was highly expressed in GC samples, confirming that FLG is up-regulated in GC tissues, which is consistent with the results in **Figure 3A** (**Figure 3C**). Semiquantitative analysis of HPA immunohistochemistry data revealed that FLG protein expression was significantly reduced in STAD compared to normal stomach tissue (**Figures 3D, E**). According to the HPA pathology scoring database (version 23.0), normal stomach tissues (n=3) exhibited strong cytoplasmic FLG staining in 100% of samples, whereas STAD tissues (n=5) predominantly showed weak or negative staining (80% low/negative, 20% moderate; *P* = 0.048, Fisher’s exact test). FLG PPI analysis was performed to explore the potential action mechanisms of FLG. The top 20 correlated genes derived from the STRING and GeneMANIA datasets were as follows: KLK5, SPINK5, KRT10, CDSN, FLG2, KRT10, CASP14, LOR, IVL, SPRR1A, DSG1, TGM1, DSC1, JUP, TCHH, PKP1, SPRR1B, DSP, PKP2, and SPINK9 (**Figures 3F, G**).

Notably, the observed FLG expression patterns exhibited platform-dependent variations requiring biological and technical contextualization (**Figure 3**). While TCGA/GTEx RNA-seq data indicated relative downregulation of FLG mRNA in STAD compared to adjacent normal tissues (**Figure 2E**), the GEO dataset GSE66229 and our *in vitro* Western blot analyses demonstrated upregulation in GC cell lines versus GES-1 and GC tissues (**Figures 3A–C**). Furthermore, HPA immuno-histochemistry revealed apparently reduced protein staining intensity in STAD compared to normal gastric mucosa (**Figures 3D, E**).

We attribute these discrepancies to the following non-mutually exclusive mechanisms: (i) Stromal contamination vs. epithelial purity: TCGA tumor specimens contain substantial stromal components (fibroblasts, immune infiltrates) that dilute the epithelial-specific FLG signal, whereas our cell line data represent pure epithelial populations without stromal noise, thereby revealing the true epithelial-derived FLG upregulation. (ii) Transcriptional-post-translational disconnect: HPA detects protein accumulation in tissue sections, whereas FLG undergoes rapid proteolytic processing and secretion. High transcriptional activity (observed in GEO/cell lines) may coexist with reduced tissue retention due to increased secretion or degradation during sample processing, explaining lower IHC detection despite elevated mRNA. (iii) Molecular subtype heterogeneity: GC demonstrates significant transcriptional heterogeneity. TCGA cohorts may be enriched in mesenchymal/subtypes with EMT-driven FLG suppression, whereas GSE66229 (Asian cohort) potentially contains more epithelial/differentiated tumors maintaining FLG expression. (iv) Platform-specific detection biases: RNA-seq (TCGA) captures total transcript pools including non-coding or degraded species, while microarray probes (GEO GPL570) and qPCR primers (our study) target protein-coding regions, potentially showing higher correlation with functional FLG protein levels observed in Western blotting. These platform-specific signatures suggest FLG exhibits context-dependent expression dynamics rather than uniform dysregulation, reinforcing its role as a differentiation-associated marker whose detection varies with tumor purity and EMT status.

### 3.2 Prognostic value of FLG in GC

**Figure 4:**
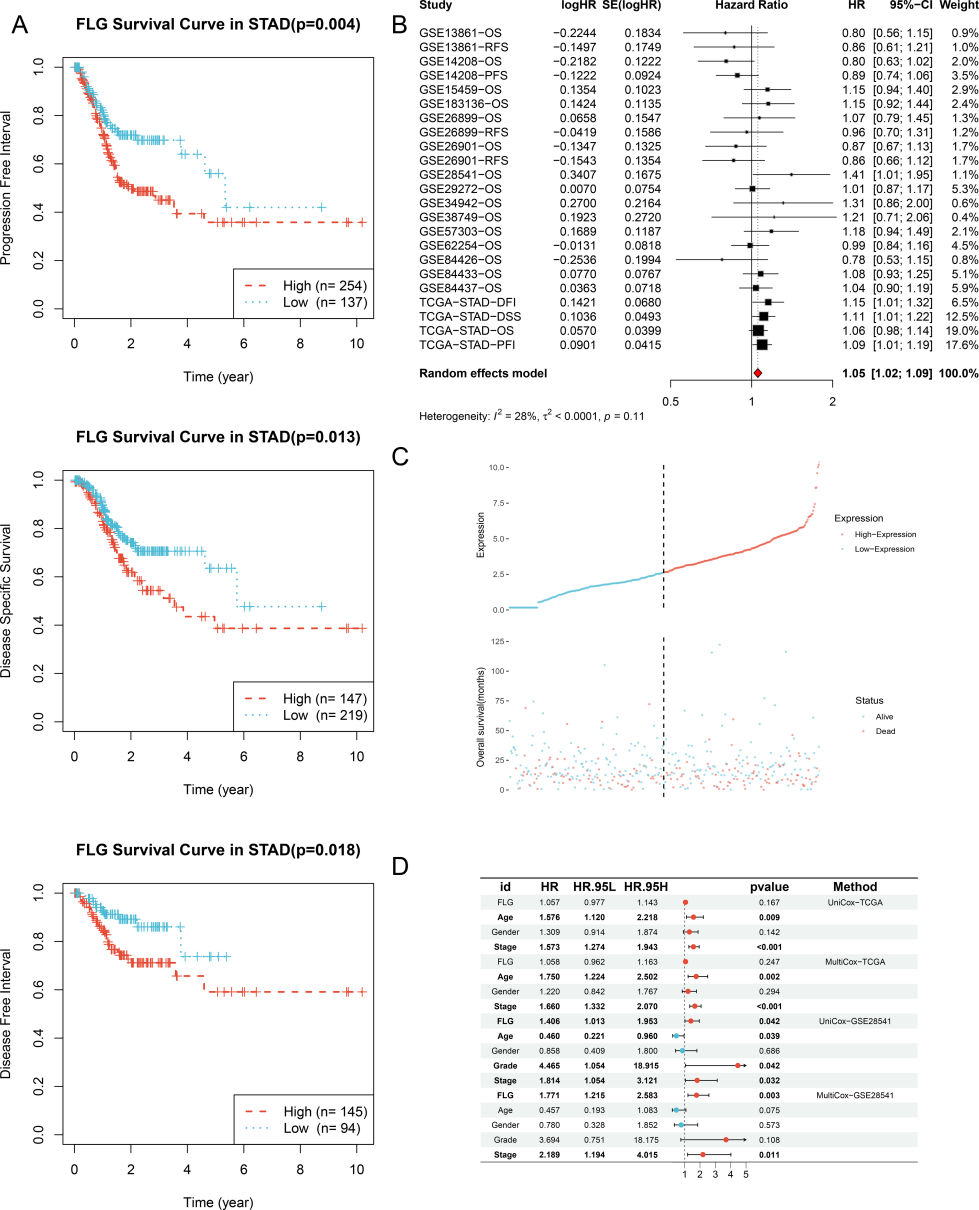
Association between FLG and survival prognosis in GC. **(A)** Correlation between FLG and different survival outcomes of GC: Progression Free Interval, Disease Specific Survival, and Disease Free Interval. **(B)** Meta-analysis of the prognostic value of FLG. (HR>1, high FLG expression is a risk factor; HR<1, high FLG expression is a protective factor.) **(C)** Association between FLG expression and clinical characteristics of GC. (The horizontal axis represents FLG expression, the vertical axis represents patients’ clinical characteristics; blue indicates low expression, and red indicates high expression). **(D)** Multivariate prognostic analysis clinicopathological factors such as age, gender, stage, and FLG expression were included, and the independent prognostic value of FLG was analyzed by multivariate Cox regression analysis.

To explore the prognostic significance of FLG in GC, we first analyzed the data of STAD in the TCGA database. The results showed that the survival probability of the high expression group declined faster over time in the Progression Free Interval (PFI) of STAD, and the statistical difference was significant (*P* = 0.004), indicating that patients with high FLG expression tend to experience disease progression. The Disease Specific Survival (DSS) curve shows the survival status of the high and low FLG expression groups after excluding other non - study disease causes caused by STAD - related diseases. The results show that the survival probability of the high expression group declines faster than that of the low expression group, indicating that high FLG expression is associated with poorer disease - specific survival outcomes, and this difference is statistically significant (*P* = 0.013). The Disease Free Interval (DFI) curve reflects the time interval from a disease - free state to disease recurrence or death in patients with high and low FLG expression. It shows that the survival probability of the high expression group declines more significantly with time, and the difference is statistically significant (*P* = 0.018), indicating that patients with high FLG expression are more likely to experience disease recurrence after treatment (**Figure 4A**). Subsequent to this, we further conducted a meta - analysis of the association between FLG and STAD survival, using a random effects model. The results showed that the combined HR was 1.05 [95% CI:1.02-1.09], *P* = 0.11, indicating that multiple studies were combined. There is a certain correlation between FLG expression and STAD survival outcomes, and the heterogeneity between studies is low (**Figure 4B**). Further presentation of FLG Overall survival curves in STAD shows that the survival status of the two groups is different, and the scatter plot on the right shows the distribution of survival (alive) and death (dead) status of patients in the high and low expression groups, reflecting the correlation between FLG expression and OS of patients (**Figure 4C**). To further investigate the clinical influencing factors of FLG expression level and disease progression or survival outcomes in STAD patients, we used uniCox and multiCox methods to analyze the HR and *P* - values of different clinical factors (such as FLG, Gender, Stage, Age, etc.) in TCGA data. The results showed that FLG expression level was closely associated with the age and stage of STAD patients, indicating that FLG expression level is an important factor affecting the prognosis of STAD patients, and high expression may indicate worse prognosis (**Figure 4D**). This result suggests that future research should further explore these significant factors, analyze the differences in FLG effects at different stages and ages, and provide clues for the prevention and treatment of GC research.

### 3.3 Association of FLG expression with clinical characteristics

**Figure 5:**
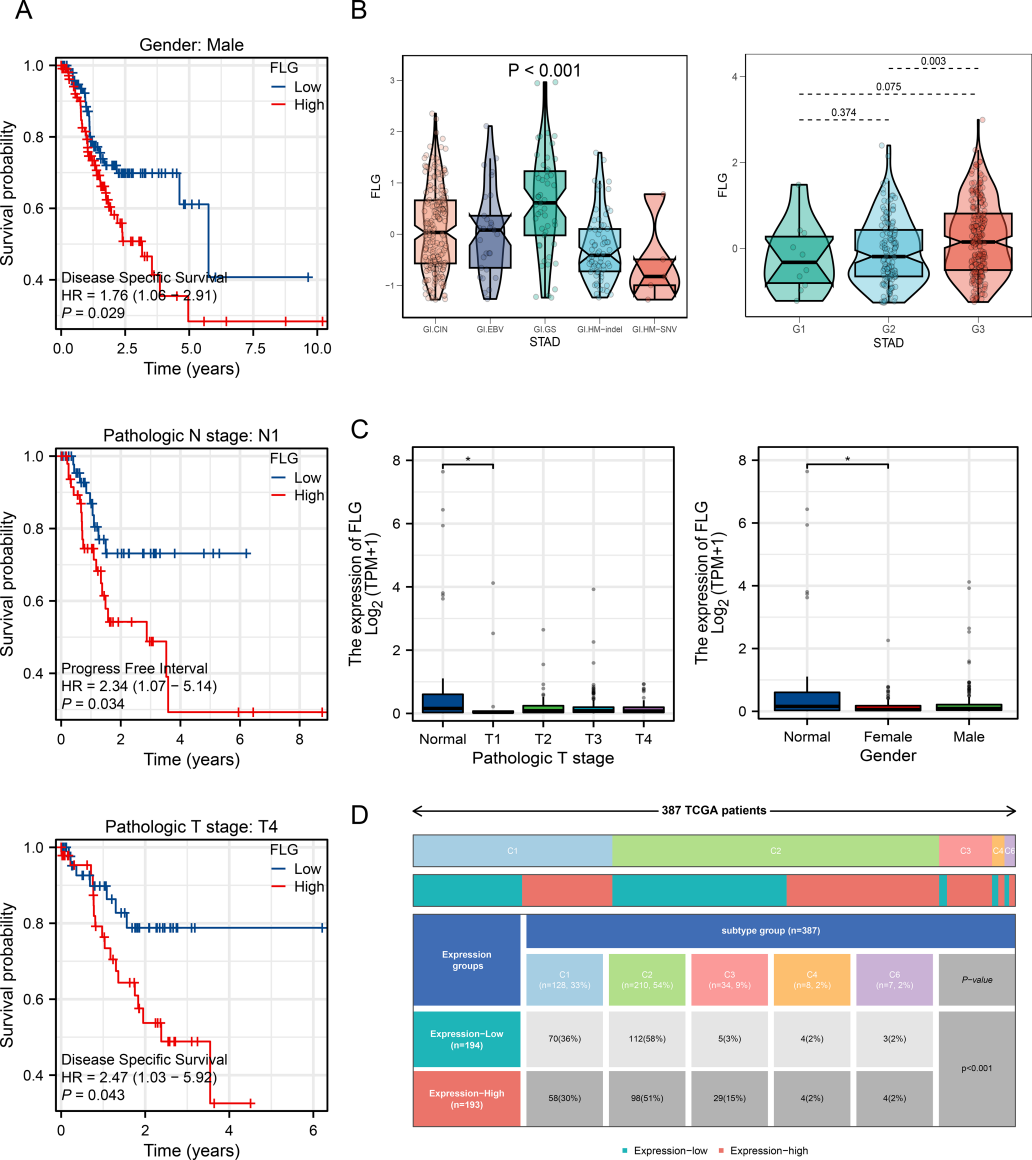
Association between FLG expression and clinicopathological features and prognosis of gastric cancer patients. **(A)** Kaplan-Meier survival curves showing the correlation between FLG and survival prognosis of GC patient subgroups. **(B)** Distribution differences of FLG expression among clinicopathological features of GC. **(C)** Validation of differences in FLG expression among clinicopathological features of GC. **(D)** Stratified analysis of FLG expression in clinical subgroups of GC.

To further explore the correlation between FLG expression and survival of GC patients, we performed clinical pathological prognostic analysis on TCGA data. **Figure 5A** shows that in male GC patients, the survival probability decreases more rapidly in the FLG high expression group, with a HR of 1.76 and a *P* value of 0.029. This suggests that high expression of FLG may be a biomarker for poor prognosis in male GC patients, and clinicians may consider FLG expression levels when evaluating the prognosis of male patients. In addition, for GC patients with pathological N1 stage, the group with high FLG expression had a shorter PFI than the group with low FLG expression, with a HR of 2.34 and a *P* value of 0.034. This indicates that high FLG expression is more strongly associated with disease progression in patients with pathological N1 stage GC. Finally, for T4 stage GC patients, the survival probability of the FLG high expression group decreased more rapidly over time, with a HR of 2.47 and a *P*-value of 0.043. In T4 stage GC patients, high expression of FLG is an indicator of poor DSS. Subsequently, we further analyzed the relationship between FLG expression and different clinical pathological groups. The violin plot shows the expression of FLG in different STAD grades (G1-G3), with significant differences in FLG expression between different grades (*P* < 0.001) (**Figure 5B**). Further analysis shows the expression of FLG in different pathological T stages (T1-T4) and gender (**Figure 5C**). The differential expression of FLG in different grade and stages is helpful for understanding the mechanism of progression. **Figure 5D** further analyzed the distribution of FLG expression groups (low/high) and subtypes in 377 TCGA patients, and there were differences in the proportion of low/high expression among different subtypes (*P* < 0.001). The association between FLG expression and subtype groups is helpful for subtyping of GC, providing a basis for precise treatment, such as exploring specific plans for patients with different subtypes and high FLG expression.

UALCAN was used to analyze the pathological characteristics, and the subgroup analysis. Logistic analysis showed that FLG expression increased in GC and correlated with gender (OR = 1.769, *P* = 0.009) (**Table 1**). Next, we used TCGA database to determine the pathological features of FLG in GC. The clinical data are presented in **Table 2**.

**Table 1:** Association between FLG expression and clinicopathologic parameters by logistic regression.

Characteristics	Total (N)	OR (95% CI)	*P* value
Pathologic T stage (T3&T4 vs. T1&T2)	367	1.312 (0.826 - 2.084)	0.249
Pathologic N stage (N1&N2&N3 vs. N0)	357	1.127 (0.719 - 1.764)	0.603
Pathologic M stage (M1 vs. M0)	355	1.110 (0.492 - 2.504)	0.802
Pathologic stage (Stage III & Stage IV vs. Stage I & Stage II)	352	0.952 (0.626 - 1.448)	0.819
Gender (Male vs. Female)	375	1.769 (1.153 - 2.713)	**0.009**
Age (> 65 vs. <= 65)	371	0.852 (0.565 - 1.284)	0.444
Histologic grade (G2&G3 vs. G1)	366	1.011 (0.288 - 3.554)	0.986

Bold values indicate statistical significance (P < 0.05).

**Table 2:** Correlation between clinicopathological variables and FLG expression.

Characteristics	Low expression of FLG	High expression of FLG	P value
N	187	188	
Pathologic T stage, n (%)			0.064
T1	15 (4.1%)	4 (1.1%)	
T2	39 (10.6%)	41 (11.2%)	
T3	78 (21.3%)	90 (24.5%)	
T4	50 (13.6%)	50 (13.6%)	
Pathologic N stage, n (%)			0.949
N0	57 (16%)	54 (15.1%)	
N1	48 (13.4%)	49 (13.7%)	
N2	36 (10.1%)	39 (10.9%)	
N3	35 (9.8%)	39 (10.9%)	
Pathologic M stage, n (%)			0.802
M0	167 (47%)	163 (45.9%)	
M1	12 (3.4%)	13 (3.7%)	
Pathologic stage, n (%)			0.552
Stage I	30 (8.5%)	23 (6.5%)	
Stage II	50 (14.2%)	61 (17.3%)	
Stage III	76 (21.6%)	74 (21%)	
Stage IV	18 (5.1%)	20 (5.7%)	
Gender, n (%)			**0.009**
Female	79 (21.1%)	55 (14.7%)	
Male	108 (28.8%)	133 (35.5%)	
Age, n (%)			0.444
<= 65	79 (21.3%)	85 (22.9%)	
> 65	108 (29.1%)	99 (26.7%)	
Histologic grade, n (%)			0.098
G1	5 (1.4%)	5 (1.4%)	
G2	78 (21.3%)	59 (16.1%)	
G3	99 (27%)	120 (32.8%)	
OS event, n (%)			0.262
Alive	119 (31.7%)	109 (29.1%)	
Dead	68 (18.1%)	79 (21.1%)	
DSS event, n (%)			**0.029**
No	139 (39.3%)	124 (35%)	
Yes	36 (10.2%)	55 (15.5%)	
PFI event, n (%)			**0.017**
No	136 (36.3%)	115 (30.7%)	
Yes	51 (13.6%)	73 (19.5%)	

Bold values indicate statistical significance (P < 0.05).

### 3.4 Enrichment analysis of FLG differential expression

**Figure 6:**
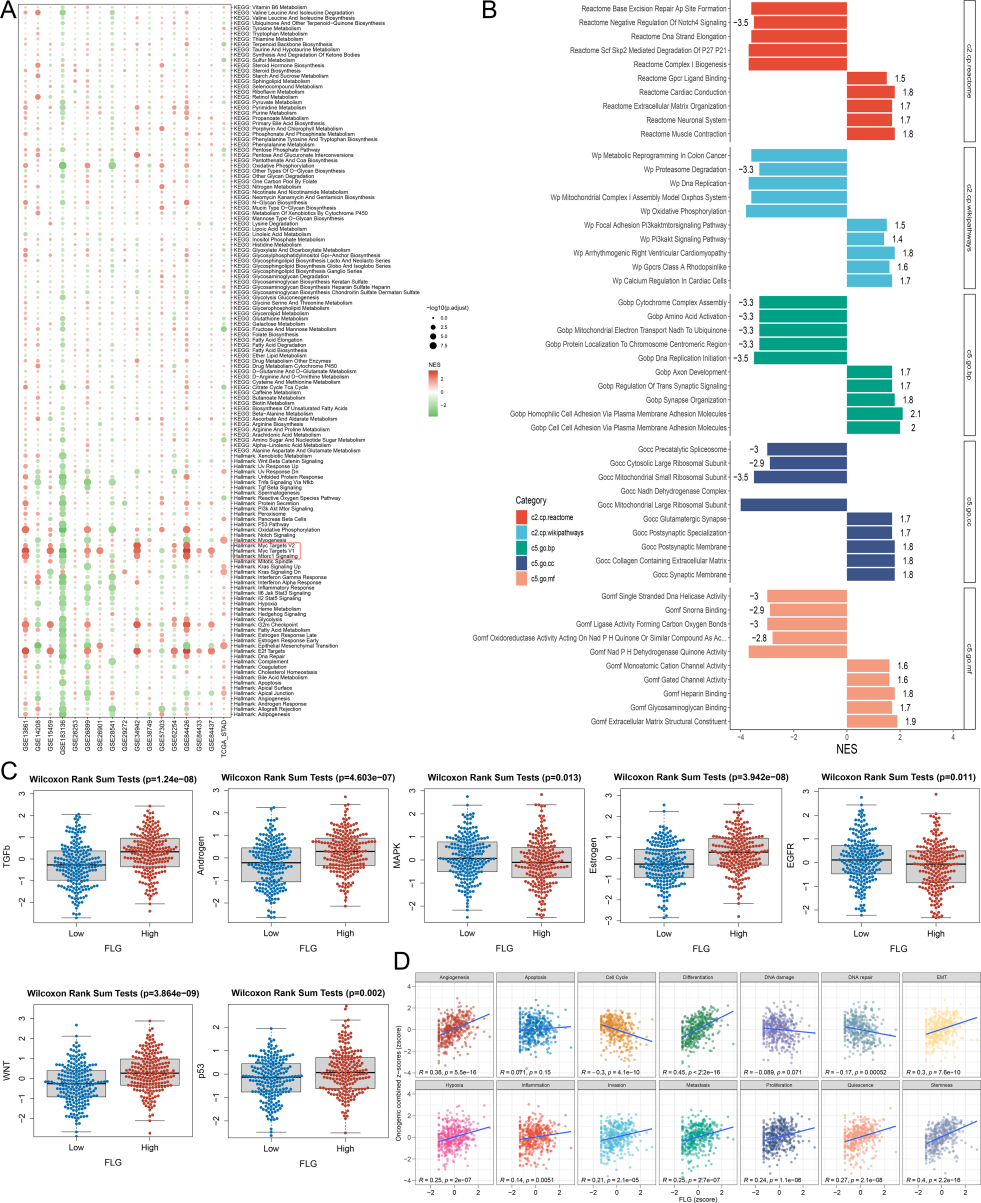
Functional enrichment analysis of the FLG gene in GC. **(A)** Heatmap of differential expression of FLG-related genes. (The horizontal axis represents samples tumor/normal, the vertical axis represents genes co-expressed with FLG, and red/green indicates high/low expression). **(B)** Functional enrichment analysis of FLG-related genes (GSEA/GO/KEGG), different colors represent different functional categories. Association analysis between FLG expression level and immune cell infiltration **(C)**, as well as immune checkpoints **(D)**.

In order to further explore the enrichment analysis of differentially expressed genes (DEGs), we conducted a comprehensive analysis of KEGG pathway, Marker enrichment, Reactome pathway, Wikipathways (WP) and Gene Ontology (GO), Biological Process (BP), Cell Component (CC), Molecular Function (MF). KEGG pathway results showed that DEGs were significantly enriched in a variety of metabolic processes, such as unsaturated fatty acid biosynthesis, citric acid cycle (TCA cycle), glycolysis/gluconeogenesis, purine/pyrimidine metabolism, and cytochrome P450 mediated exogenous substance metabolism, as well as signal related pathways including TGF-*β* signaling and Wnt/*β*-catenin signaling. Marker enrichment analysis revealed that the pathways related to stress response (reactive oxygen species pathway, down/up regulation of ultraviolet response, unfolded protein response), signal transduction (through TNF-*α* signal transduction of NF-κb) and cell function (spermatogenesis, exogenous substance metabolism) were activated, which further supported the role of stress adaptation and signal regulation in the biological processes studied (**Figure 6A**). Reactome and Wikipaths analysis supplemented the above findings by identifying enrichment pathways related to DNA repair (base excision repair, DNA strand elongation), cell cycle regulation (scf-skp2 mediated *p*27/*p*21 degradation), mitochondrial function (oxidative phosphorylation, mitochondrial complex I assembly) and cancer-related metabolic reprogramming (metabolic reprogramming in colon cancer, PI3K-Akt signaling pathway). The standardized enrichment score (NES) of key pathways ranged from 1.4 to 1.9, indicating significant enrichment. Notably, while metabolic reprogramming pathways (TCA cycle, glycolysis) showed the highest NES scores, the EMT pathway exhibited the strongest phenotypic correlation with FLG expression (R = 0.42, *P* < 0.001, **Figures 6B–D**), directly linking FLG to invasive progression. GO enrichment analysis showed that DEGs were mainly located in cellular components. In addition, Wilcoxon rank sum test showed that there were significant differences in the expression of key pathway markers (TGF-*β*, EGFR, Wnt) between FLG high expression group and FLG low expression group (*P* < 0.05), and there was a positive correlation between EMT signature, Differentiation, Angiogenesis, Stemness and FLG (R = 0.3~0.45, *P* < 0.05), consistent with FLG’s structural role in epithelial integrity. Overall, these enrichment results indicate that differentially expressed genes are mainly involved in metabolic reprogramming, stress response, signal transduction and DNA repair, which provide important insights into the potential molecular mechanisms of FLG expression related phenotypes.

### 3.5 Genetic variation and tumor mutational burden analysis of FLG in GC

**Figure 7:**
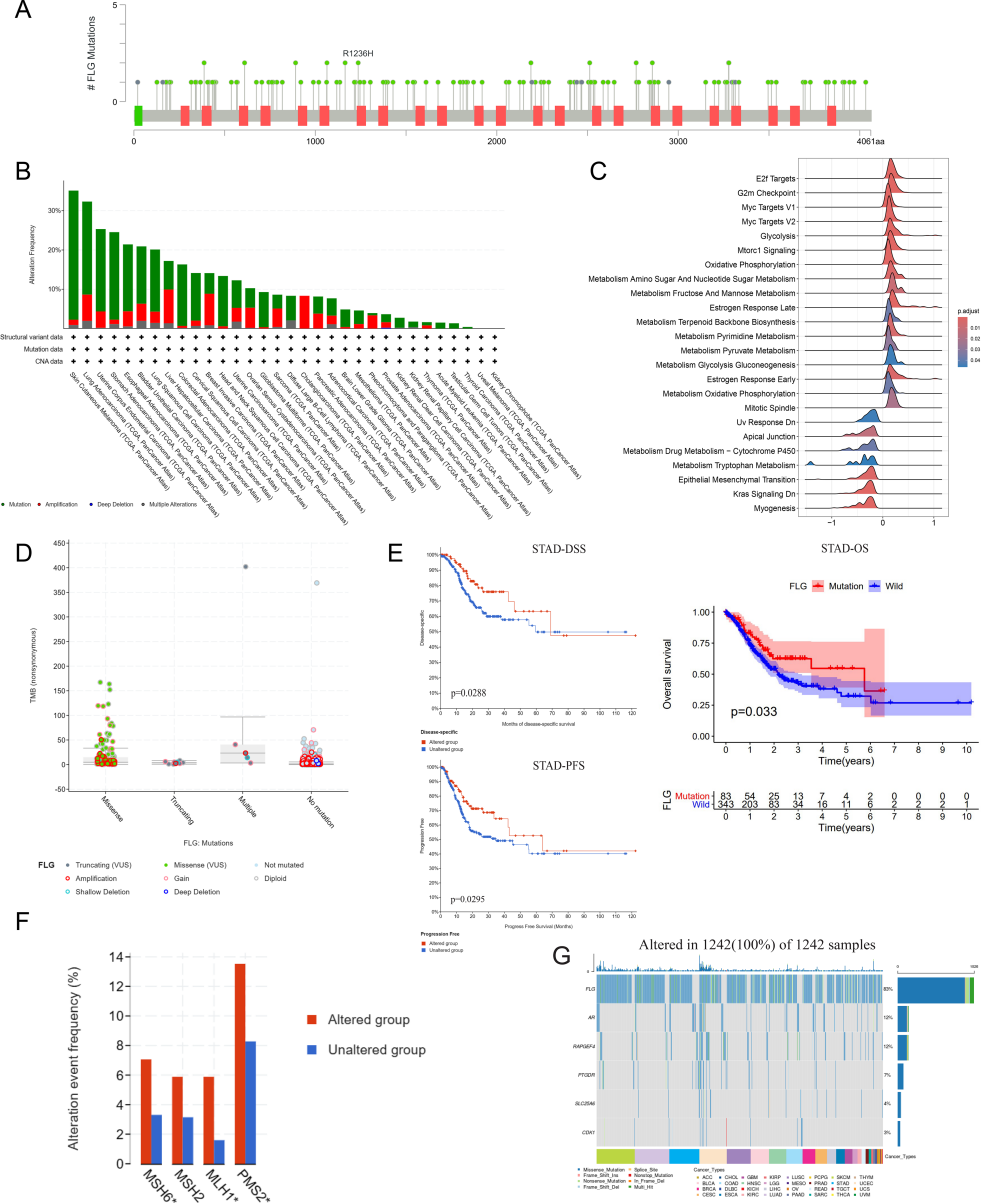
Genetic variations of the FLG in GC. **(A)** Distribution of mutation sites in the FLG. **(B)** Association between FLG mutation types and protein domains. **(C)** Functional pathway enrichment associated with FLG mutations. **(D, E)** Association of FLG mutations with tumor mutation burden (TMB) and prognosis. **(F, G)** Association of FLG mutations with DNA mismatch repair (MMR)-related genes.

**Figure 8:**
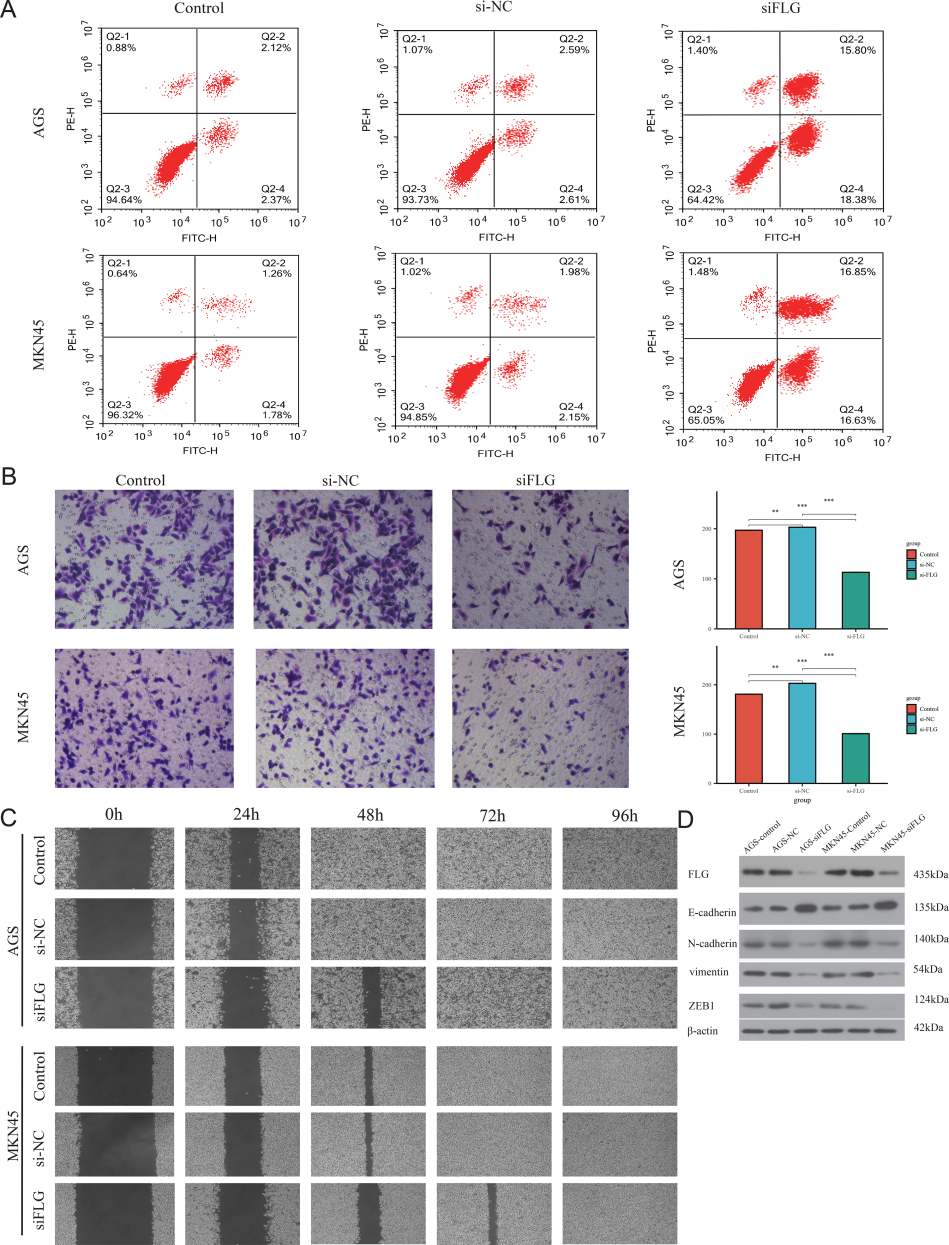
The impact of FLG on the malignant phenotype of GC. **(A)**Flow cytometry detected GC cell apoptosis. **(B)** Transwell assays detected invasion and migration of GC cells. **(C)** Wound-healing assays detected dynamic effect of GC cells. **(D)** EMT signaling pathway was detected using WB. *P < 0.05, **P < 0.01.

To investigate genetic alterations of FLG in GC, we used cBioPortal and GSCALite to analyze gene alterations and mutational events. The results showed that there were multiple high-frequency mutation sites in the coding region of the FLG gene, and hotspot mutations such as R1236H were concentrated in the functional domain (such as the domain’s coding region), suggesting that they may affect protein function (**Figure 7A**). The mutation types and sites in FLG were shown in **Figure 7B**. SNV Oncoplot, CNV, mRNA RSEM, methylation, and gene expression were analyzed by means of GSCALite. The highest frequency of FLG alterations was observed in the SKCM, LUAD, UCEC, STAD, and ESCC. To further analyze the enrichment of functional pathways related to FLG mutation, we used GSEA/KEGG enrichment analysis to explore the metabolic pathways and signaling pathways activated following FLG mutation. The results showed that FLG mutation was enriched in specific pathways (such as “E2F targets or EMT”), suggesting that FLG mutation may reprogram metabolic/immune pathways (**Figure 7C**). However, notably, patients with FLG mutations exhibited significantly higher TMB (**Figure 7D**) and better survival outcomes (OS, DSS, PFS; **Figure 7E**) than wild-type patients. This apparent contradiction is explained by the fact that FLG mutations in STAD are predominantly loss-of-function (LOF) variants (**Figure 7B**) that may abrogate the pro-invasive function of wild-type FLG (as demonstrated in **Figure 8**), while concomitantly serving as markers of microsatellite instability (MSI) or enhanced immunogenicity (evidenced by elevated DNA repair gene expression in **Figure 7F**), thereby paradoxically improving prognosis despite pathway enrichment. Further analysis of 1242 pan-cancer samples showed that FLG gene had the highest mutation frequency in a variety of species, up to 80%; The second highest was the AR gene, with a mutation frequency of 12%, which is conducive to exploring FLG as a potential mechanism or biomarker of cancer occurrence and development (**Figure 7G****).**

### 3.6 Correlation with immune cells of FLG

**Figure 9:**
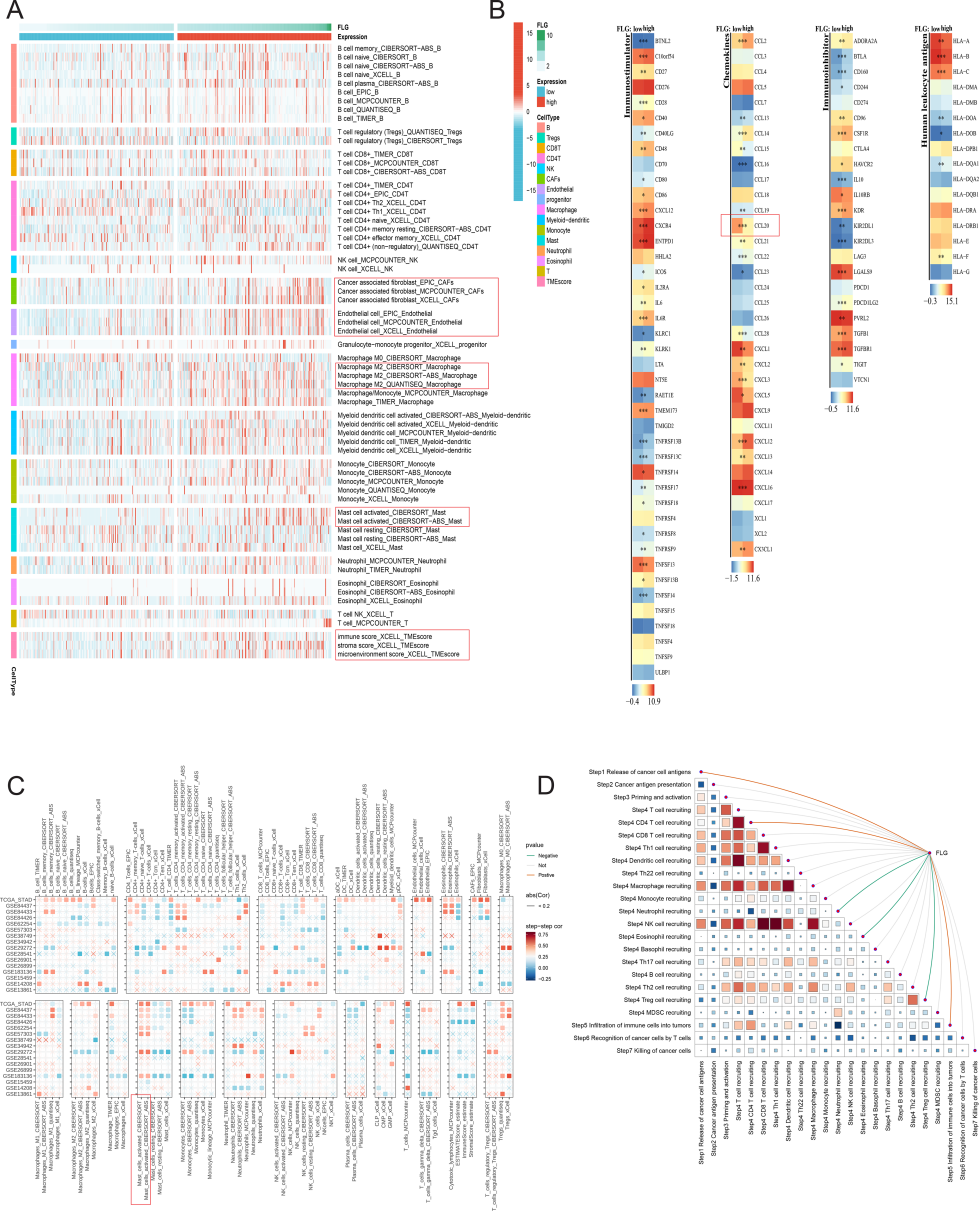
Correlation between FLG and immune cells/immune microenvironment in GC. **(A)** Heatmap of FLG-related gene expression and functional clustering (The horizontal axis represents samples tumor/normal, the vertical axis represents genes co-expressed with FLG, red/blue indicates high/low expression, and functional clustering is shown on the right). **(B)** Correlation between FLG and immune cell markers. **(C)** Heatmap of the correlation between FLG and immune checkpoints, as well as the tumor microenvironment. **(D)** Correlation between FLG and the molecular network of the tumor microenvironment.

To explore the correlation between FLG expression level and different immune cell infiltration, we first evaluated the immune cell infiltration in TCGA STAD samples using multiple algorithms (including CIBERSORT-ABS, Quantiseq, Mcpcount, Xcell, EPIC, TIMER, etc.). The results showed that cancer-related fibroblasts, endothelial cells, macrophages and other immune cells were highly expressed in some samples, and the infiltration characteristics were more prominent in FLG high expression samples (**Figure 9A**). **Figure 9B** focuses on the correlation between different molecular characteristics (including immune checkpoint molecules, chemokines, immunosuppressive molecules and human leukocyte antigen-related molecules) and FLG expression levels. The heat map showed that the expression level of FLG was higher in the high expression group, such as certain immune checkpoint molecules, however some chemokines were more strongly correlated in the low expression group of FLG, which provided the basis for further revealing the immune regulation mechanism and the interaction between molecular characteristics and immune cell infiltration.

Further, GSE STAD samples verified that the high expression level of FLG was significantly correlated with the infiltration of macrophages and other immune cells (**Figure 9C**), suggesting that they might participate in common biological processes or be affected by similar regulatory mechanisms, providing clues for the subsequent screening of key regulatory genes. **Figure 9D** shows the enrichment analysis results of immune-related pathways, showing the correlation between FLG and various immune-related pathways through network visualization. The results showed that FLG was associated with immune cell activation (such as T cell and macrophage activation related pathway), chemotaxis (such as chemokine mediated cell chemotaxis pathway), differentiation (such as immune cell differentiation related pathway), and immune response regulation (such as innate response and immune response pathway), which fully indicated that FLG was widely involved in a variety of immune process-related pathways and played a role in multiple levels of immune microenvironment. The above analysis reveals that FLG is involved in a variety of key immunobiological processes, providing a more comprehensive perspective for in-depth analysis of FLG mediated regulation mechanism.

### 3.7 Construction of regulatory network for FLG-associated ceRNA

**Figure 10:**
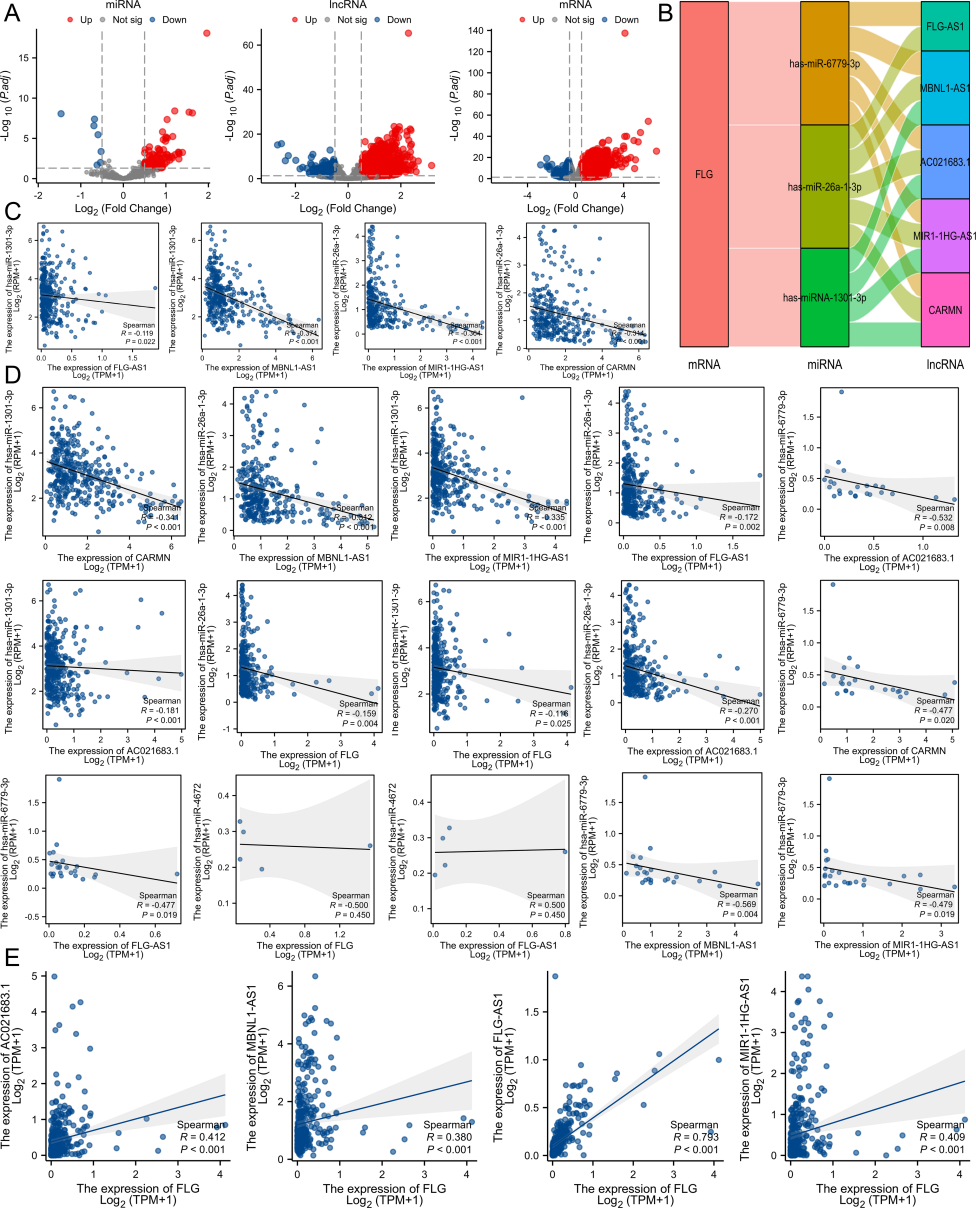
Prediction the ceRNA networks of FLG in GC. **(A)** The volcano plots describe DElncRNAs, DEmiRNAs, and DEmRNAs in GC with TCGA database. **(B)** FLG-related ceRNA regulatory network were shown by Sankey diagram. **(C–E)** miRNAs or lncRNAs correlated with FLG were shown by Scatter plots. LncRNAs correlated with miRNAs were shown by Scatter plots.

Using screening criteria of |log_2_FC|≥1.0 and adjusted *P* < 0.05, we identified differentially expressed lncRNAs, miRNAs, and mRNAs from TCGA-STAD dataset. Correlation threshold |R|≥0.4 (*P* < 0.05) was applied to construct the ceRNA network shown in **Figure 10**.

Increasing evidence demonstrates the regulatory effects of ceRNA networks in GC. Volcano plots were used to describe DElncRNAs, DEmiRNAs, and DEmRNAs in STAD using TCGA (**Figures 10A, B**). Four human-derived FLG, most related miRNAs (miR-6779-3p, miR-4672-3p, miR-26a-1-3p, and miR-1301-3p) and five top-related lncRNAs (FLG-AS1, MBNL1-AS1, AC021683.1, MIR1-1HG-AS1, and CARMN) are shown. miRNAs (miR-6779-3p, miR-26a-1-3p, and miR-1301-3p) were found to be negatively correlated with FLG expression. The lncRNAs (FLG-AS1, MBNL1-AS1, AC021683.1, MIR1-1HG-AS1 and CARMN) were verified to positively correlate with FLG expression. Scatter plots were used to display the expression of lncRNAs and miRNAs (**Figures 10C–E**).

### 3.8 Knockdown of FLG inhibited GC cells proliferation, migration and invasion

Based on the significant enrichment of EMT pathways in our GSEA analysis (**Figures 6C, D**) and the clinical correlation between high FLG expression and invasive stage (T4/N1) poor prognosis (**Figure 5A**), we hypothesized that FLG promotes GC progression through EMT activation.

**Figure 11:**
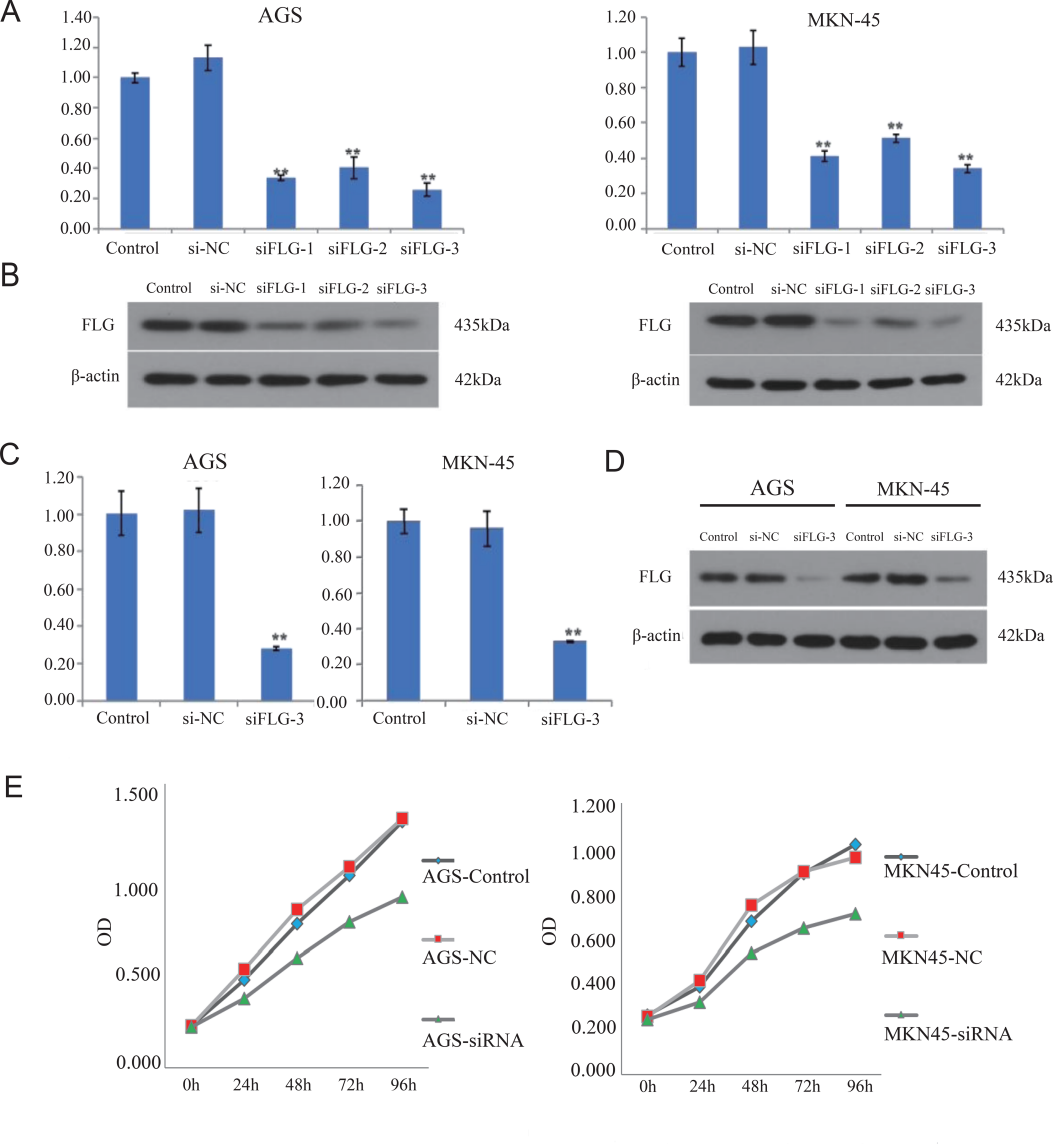
The effects of FLG in GC cells. **(A–D)** siRNA–FLG was evaluated in AGS and MKN45 GC cells with qRT-PCR and western blotting. **(E)** CCK8 assays detected GC cell proliferation.

To validate this mechanism and minimize off-target effects, three independent siRNAs targeting distinct FLG regions were evaluated. qRT-PCR and Western blotting confirmed that all three siRNAs achieved significant FLG knockdown, with siFLG-3 showing the highest efficiency (**Figures 11A–D**). Importantly, preliminary experiments with siFLG-1 and siFLG-2 produced qualitatively similar inhibitory effects on cell proliferation and migration, confirming phenotype specificity. Given its superior knockdown efficiency, siFLG-3 was selected for subsequent comprehensive phenotypic assays.

In addition, we used a CCK8 kit to detect changes in cell activity in the negative carrier group and transfected siFLG-3 group at different time points. The results showed that interference with FLG gene expression significantly reduced cell proliferation (**Figure 11E**). Flow cytometry revealed that FLG knockdown inhibited GC cell apoptosis (**Figure 8A**). Moreover, transwell chamber experiments demonstrated that the invasion ability of GC was weakened after FLG was knocked out (**Figure 8B**). Furthermore, the healing ability of GC cells that interfered with FLG expression was significantly weakened in the cell scratch experiments (**Figure 8C**). The EMT signaling pathway was detected using WB. FLG promoted E-cadherin gene expression and inhibited N-cadherin, vimentin, and ZEB1 expression, suggesting that FLG promotes EMT signaling. (**Figure 8D**).

## 4 Discussion

In this study, we demonstrate that wild-type FLG overexpression drives GC progression through EMT activation, establishing a previously unrecognized oncogenic mechanism distinct from the loss-of-function mutational paradigm observed in other malignancies. Our multi-omics analysis reveals that FLG serves as a robust prognostic biomarker, with high expression specifically associated with poor outcomes in male patients and advanced-stage (T4/N1) GC, underscoring its clinical relevance in high-risk subgroups.

While metabolic pathways exhibited prominent enrichment scores, we prioritized EMT based on three converging lines of evidence. First, the PPI network revealed FLG’s intimate association with epithelial structural proteins (desmosomal components DSG1/DSC1/JUP, keratins), suggesting its oncogenic role manifests through cell-cell adhesion disruption rather than metabolic modulation (7). Second, FLG’s prognostic significance was most pronounced in T4/N1 stages-clinical states characterized by invasion/metastasis, precisely the EMT-driven phenotypes. Finally, as a filament-associated protein fundamental to epithelial barrier integrity, FLG’s dysregulation would logically compromise adhesion and enhance migratory capacity, whereas metabolic reprogramming would require secondary metabolomic validation beyond the scope of this mechanistic study. Thus, the convergence of network topology, invasive clinical phenotypes, and gene ontology uniquely positioned EMT as the most biologically plausible mechanism for FLG-driven GC progression.

Our findings regarding FLG mutations align with yet mechanistically extend Fu et al.’s report that GC patients carrying FLG mutations exhibit superior survival (22). While we initially observed pathway enrichment in metabolic/immune processes, the association of FLG mutations with better survival and higher TMB indicates these are likely passenger mutations occurring in MSI-high tumors with defective mismatch repair (evidenced by elevated MSH6/MLH1/PMS2 expression). Given that wild-type FLG promotes EMT and invasion, loss-of-function mutations may functionally inhibit rather than promote progression, while the hypermutated phenotype confers enhanced immunogenicity-a dual explanation reconciling this apparent paradox. Thus, FLG mutation status likely serves as a prognostic biomarker for immunogenic tumor subtypes rather than a driver of malignancy.

Similarly, the apparent contradictions in FLG expression patterns across TCGA (downregulation), GEO GSE66229 (upregulation), HPA (reduced protein), and our cell line experiments reflect the multifaceted nature of filaggrin biology rather than data inconsistencies. Stromal contamination in bulk TCGA tissues likely obscures epithelial-specific FLG signals, whereas our cell line data represent pure malignant epithelial populations revealing intrinsic upregulation. Furthermore, FLG undergoes rapid proteolytic processing and secretion; consequently, high transcriptional activity may coincide with reduced protein accumulation in tissue sections (HPA IHC) due to accelerated turnover or secretion into the gastric lumen (6). Ethnic variations between cohorts (Asian vs. Western populations) and molecular subtype differences (intestinal-type vs. diffuse-type GC) additionally contribute to expression divergence. These discrepancies underscore that FLG serves as a dynamic, context-dependent biomarker requiring stratification by tumor purity, EMT status, and ethnic background. Finally, technical normalization differences contribute to expression divergence: TCGA RNA-seq employs TPM normalization quantifying transcript abundance, whereas GEO microarray utilizes RMA normalization optimizing signal-to-noise ratios, introducing platform-specific quantitative biases.

Our findings reveal that wild-type FLG overexpression drives GC progression through EMT activation-a mechanism distinct from the mutational paradigm observed in other carcinomas. In early-onset breast cancer, FLG mutations represent structural protein alterations contributing to genomic instability (18), whereas in GC, we demonstrate that intact FLG protein promotes invasion, suggesting an expression-driven oncogenesis. This discrepancy likely reflects tissue-specific functional requirements: while breast epithelium relies on FLG for structural maintenance, gastric mucosa utilizes FLG in dynamic epithelial renewal processes that can be co-opted for metastatic dissemination.

Consistent with studies linking FLG dysregulation to HPV-induced cervical carcinogenesis (23, 24), we observed FLG overexpression in malignant gastric epithelium; however, we extend these observations by functionally validating its causal role in invasion through siRNA-mediated knockdown and EMT marker analysis. In contrast to skin malignancies, where FLG downregulation correlates with cutaneous T-cell lymphoma progression (19, 20) and where filaggrin overexpression in melanoma paradoxically associates with immune evasion and poor prognosis (25–29), GC cells utilize FLG to enhance intrinsic migratory capacity via E-cadherin/N-cadherin switching. This suggests FLG’s oncogenic potential manifests through distinct tissue-specific modalities: immune modulation and barrier dysfunction in skin versus direct cytoskeletal remodeling and EMT induction in gastric epithelium. These contextual differences underscore the necessity of cancer-type-specific interpretation of FLG biomarker data.

Our analysis also revealed that FLG is highly mutated and significantly affects GC prognosis and immune infiltration, including CNV and missense mutations, findings that partially agree with previous studies (30). Notably, FLG was strongly correlated with NK cell infiltration, and the NK cell enrichment scores in high-FLG-expressing cells were significantly elevated. Increasing evidence demonstrates that improved NK cell infiltration or function significantly benefits patient survival (31–33). To explore the potential immunomodulatory effects of FLG on GC, we identified lymphocytes, immunomodulators, chemokines, receptors, and MHC molecules associated with FLG. These results indicate that up-regulation of FLG in GC is linked to immune cell infiltration; however, the specific mechanism by which FLG-overexpressing tumors interact with NK cells and other immune populations requires further investigation.

Furthermore, growing evidence supports the regulatory role of lncRNA-miRNA-mRNA networks in the FLG ceRNA axis in cancer. Based on our results, we constructed a ceRNA regulatory network predicting that FLG may regulate several critical pathways in GC. The use of multiple independent siRNA sequences targeting different FLG regions, all producing concordant phenotypic effects, minimizes concerns regarding off-target artifacts and strengthens the conclusion that FLG specifically drives GC cell proliferation and EMT.

The present study has several limitations. First, while FLG has been shown to have high prognostic value in patients with GC, and its biological function and regulatory signaling pathways have been tentatively validated at the cellular level, the understanding of real case samples and detailed regulatory mechanisms is currently limited. Second, more detailed *in vitro* testing techniques using complementary approaches (e.g., metabolomics, single-cell sequencing) will further help in elucidating the therapeutic role of FLG in patients with GC. Finally, additional clinical data are needed to validate and elucidate the expression and role of FLG in GC and other gastrointestinal malignancies.

## 5 Conclusion

This study identifies FLG as a novel prognostic biomarker and therapeutic target in GC. We demonstrate that wild-type FLG drives malignant progression through EMT activation, while FLG mutations paradoxically mark immunogenic subtypes with better survival. These findings establish a context-dependent oncogenic mechanism distinct from other malignancies and provide a foundation for precision diagnostics and individualized immunotherapy strategies in GC.

## Data Availability

Publicly available datasets were analyzed in this study. The data presented in the study are deposited in the Gene Expression Omnibus (GEO) repository, accession number GSE66229 (https://www.ncbi.nlm.nih.gov/geo/query/acc.cgi?acc=GSE66229), and The Cancer Genome Atlas (TCGA) repository (https://portal.gdc.cancer.gov). The original experimental data presented in the study are included in the article and **Supplementary Material**. Further inquiries can be directed to the corresponding authors.
