# Supplementary figures and images for: Filaggrin as a potential biomarker in gastric cancer: insights from multi-omics analysis and experimental validation
Source: Front Immunol. 2026 May 8;17:1742982. doi: 10.3389/fimmu.2026.1742982 (PMC13194527; doi:10.3389/fimmu.2026.1742982)

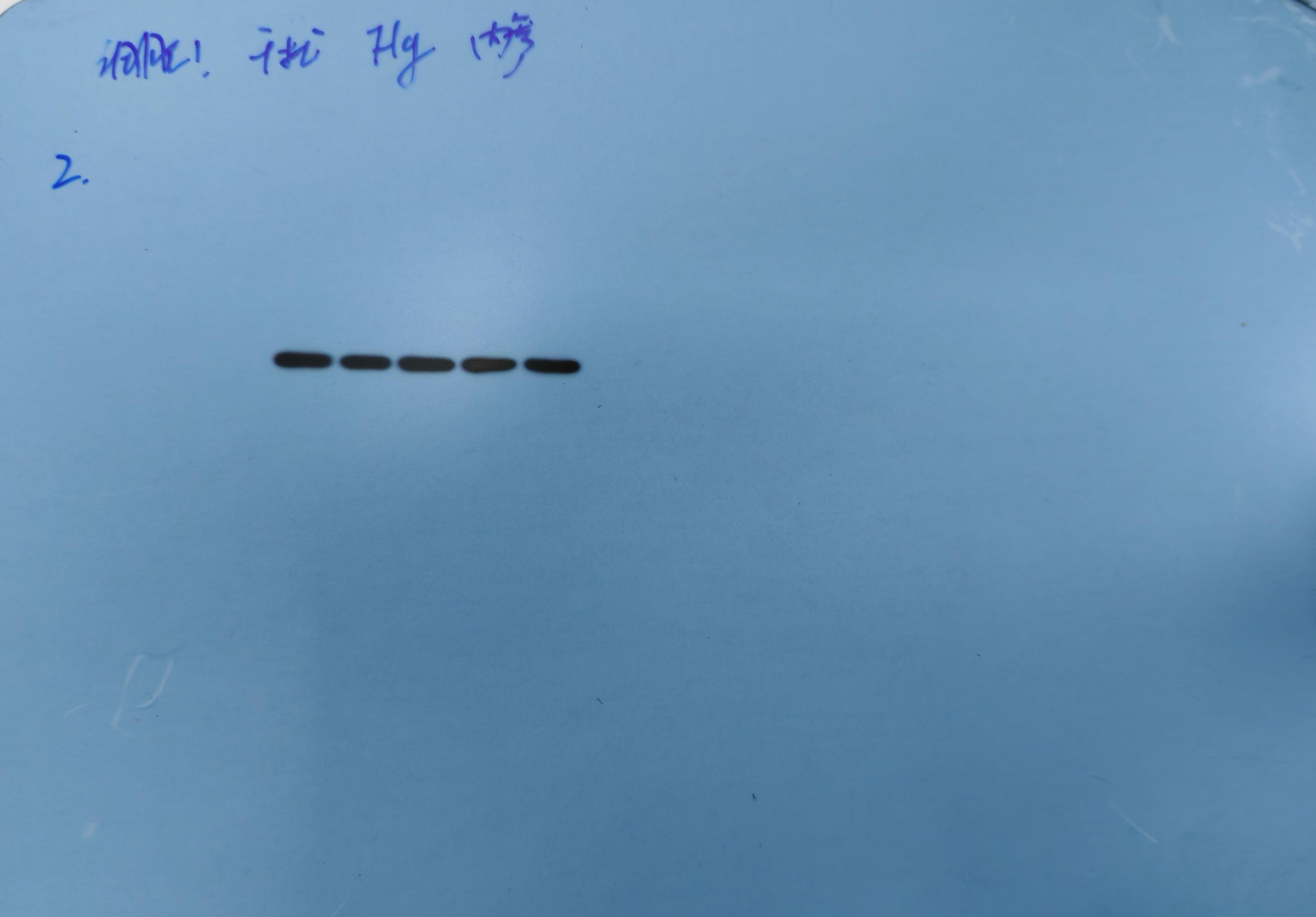

Supplement: Supplementary file 1 [file DataSheet1.zip › Supplymentary/Figure 10. B sh-RNA FLG expression in AGS cell b-actin.jpg]

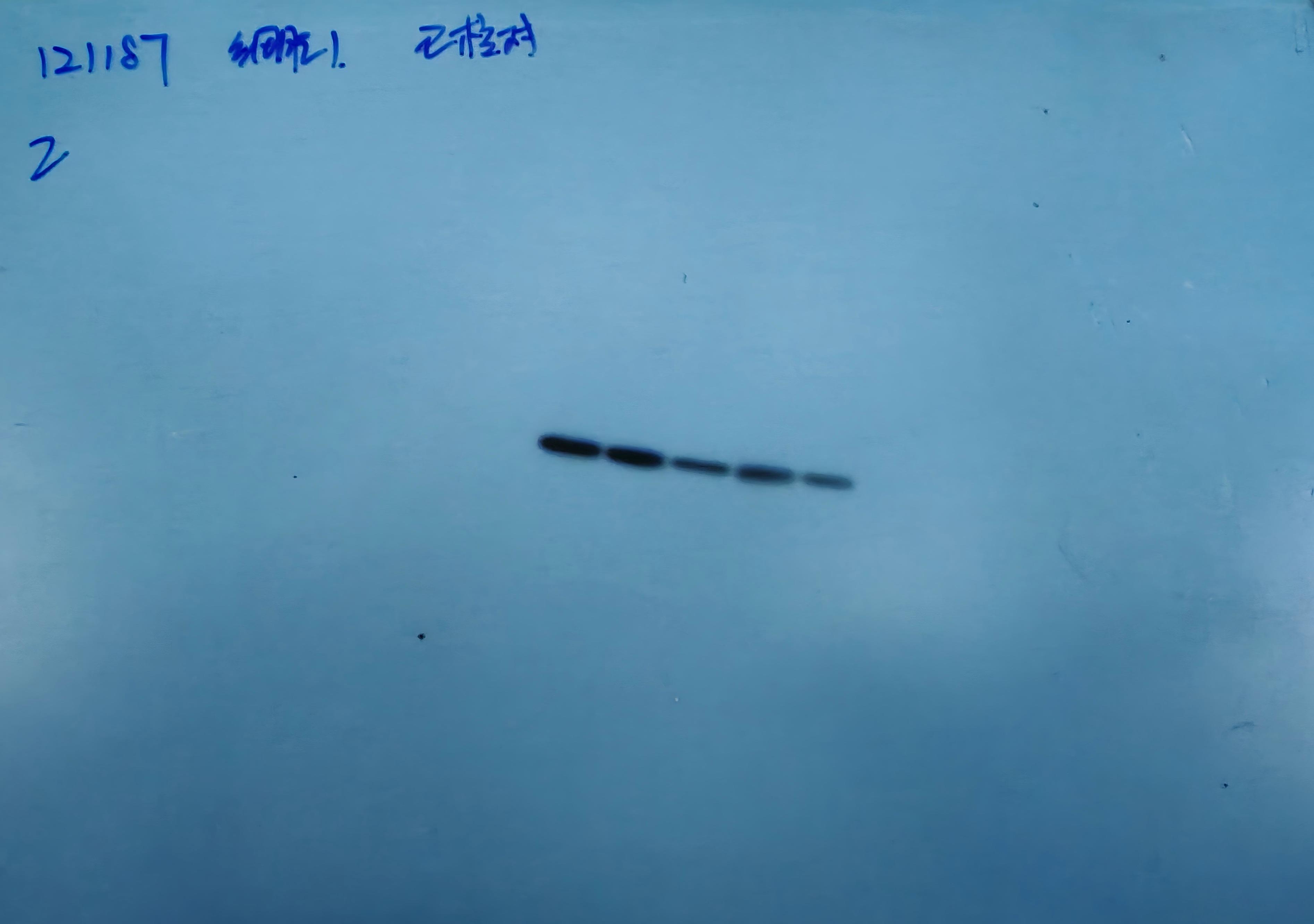

Supplement: Supplementary file 1 [file DataSheet1.zip › Supplymentary/Figure 10. B sh-RNA FLG expression in AGS cell.jpg]

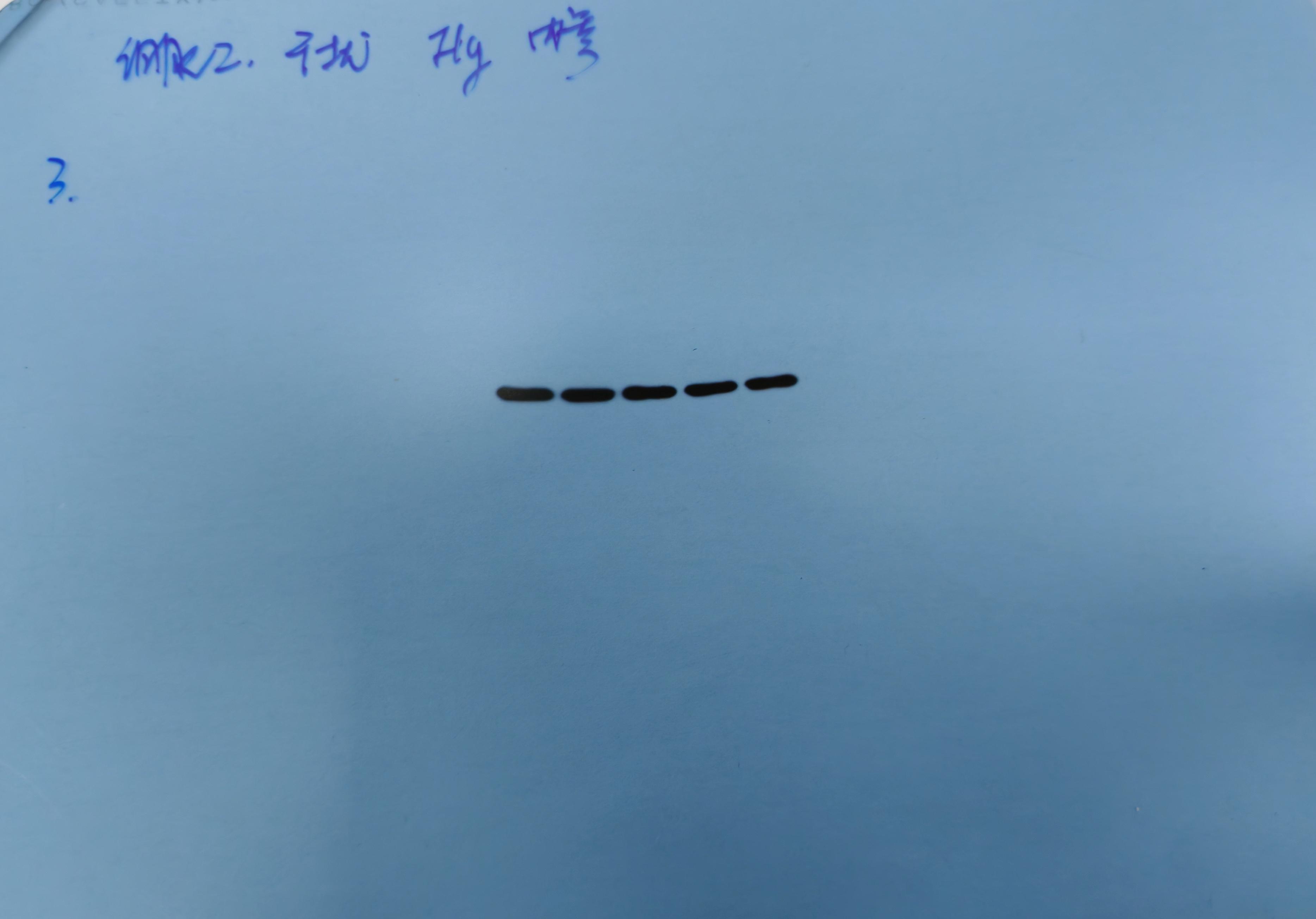

Supplement: Supplementary file 1 [file DataSheet1.zip › Supplymentary/Figure 10. B sh-RNA FLG expression in MKN-45 cell b-actin.jpg]

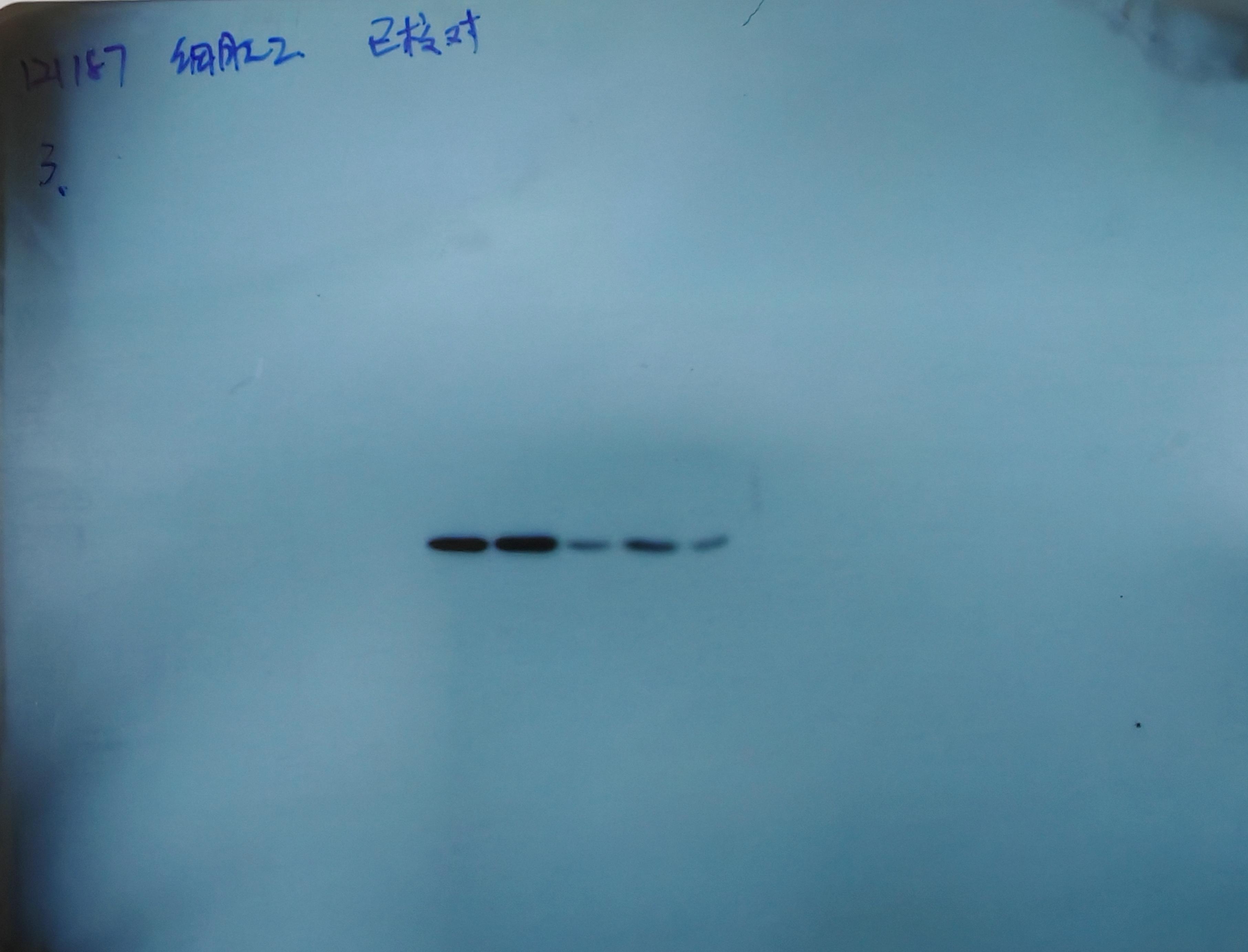

Supplement: Supplementary file 1 [file DataSheet1.zip › Supplymentary/Figure 10. B sh-RNA FLG expression in MKN-45 cell.jpg]

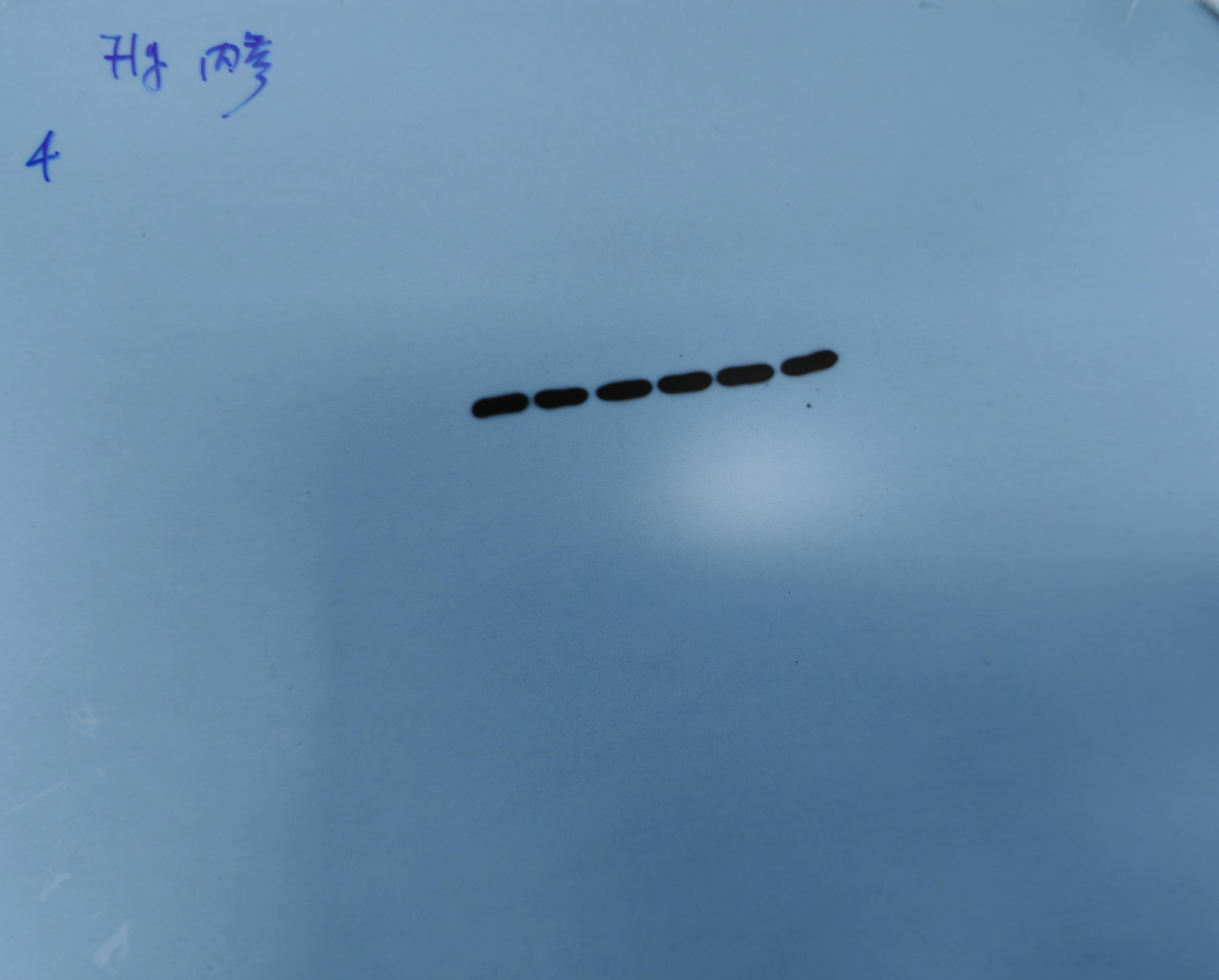

Supplement: Supplementary file 1 [file DataSheet1.zip › Supplymentary/Figure 10. D FLG expression in AGS & MKN-45 cells b-actin.jpg]

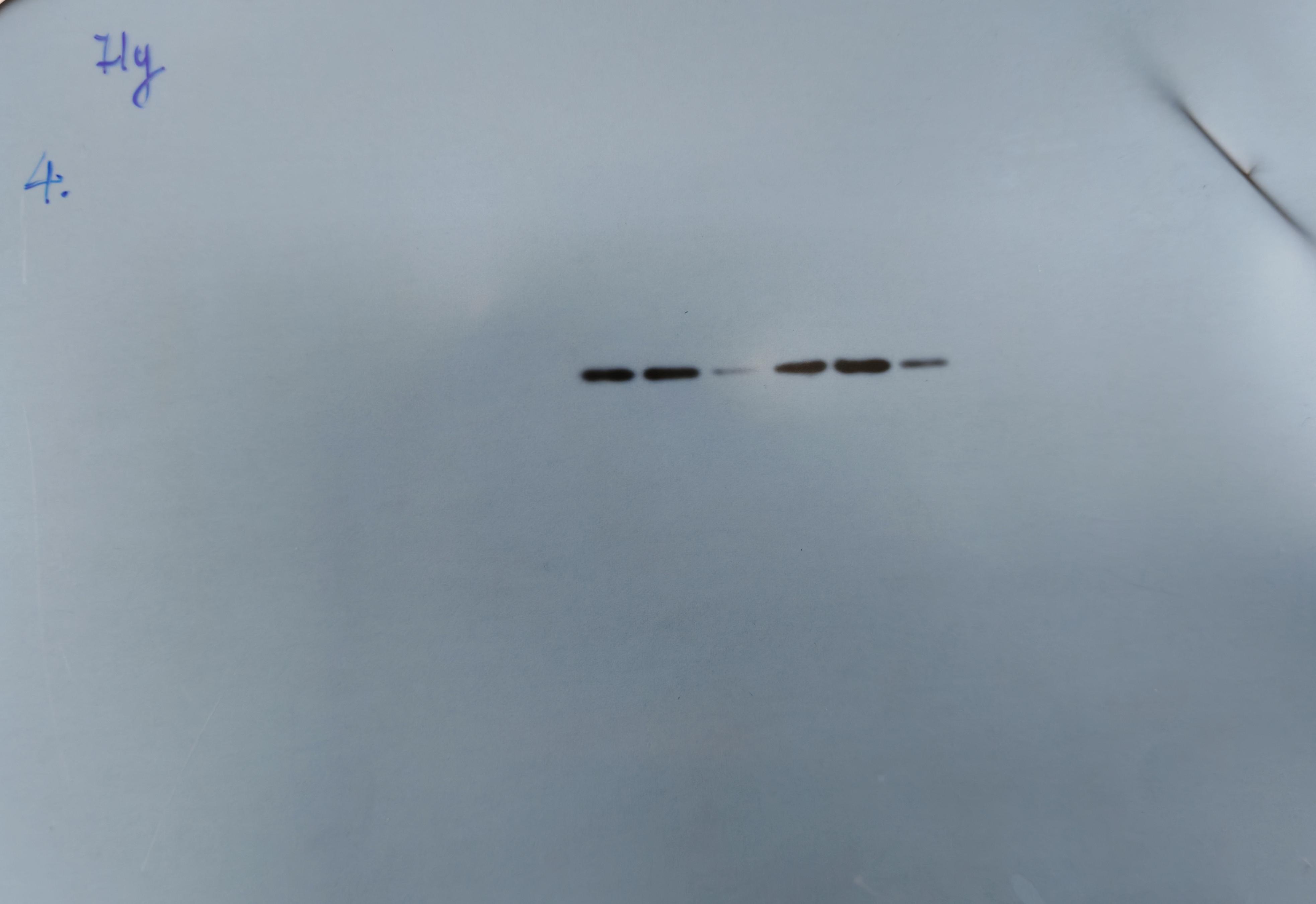

Supplement: Supplementary file 1 [file DataSheet1.zip › Supplymentary/Figure 10. D FLG expression in AGS & MKN-45 cells.jpg]

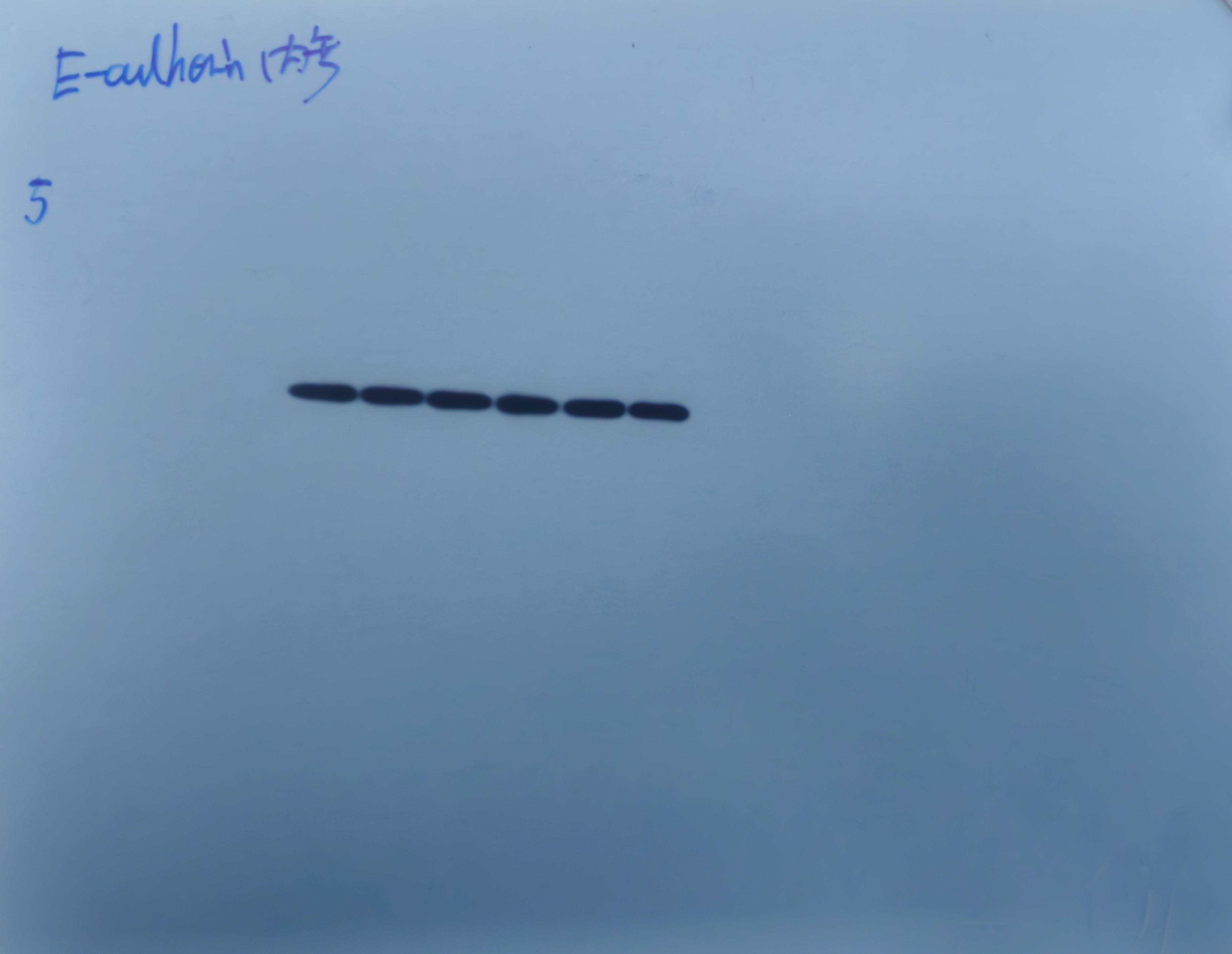

Supplement: Supplementary file 1 [file DataSheet1.zip › Supplymentary/Figure 11. D E-cadherin expression in cells b-actin.jpg]

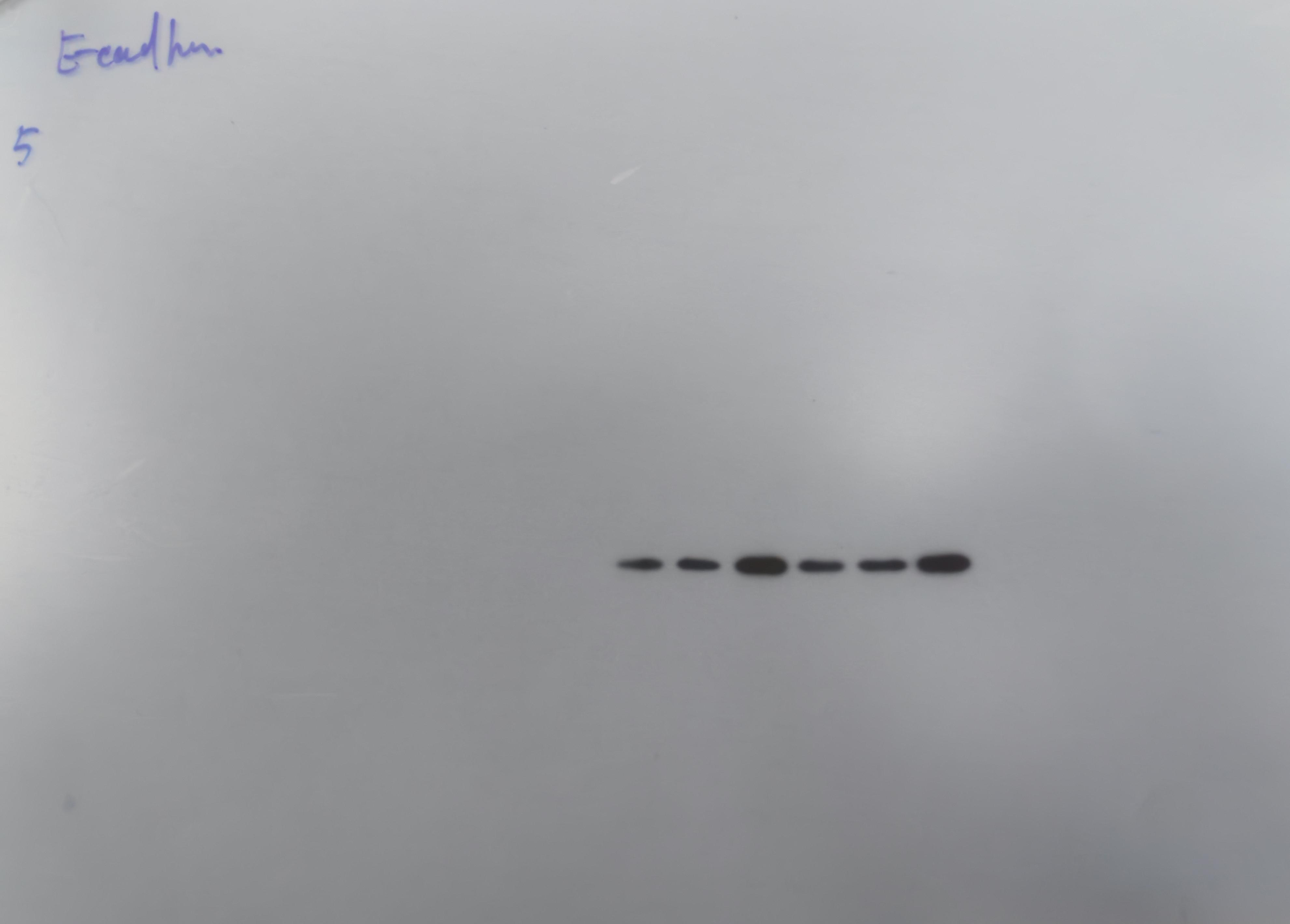

Supplement: Supplementary file 1 [file DataSheet1.zip › Supplymentary/Figure 11. D E-cadherin expression in cells.jpg]

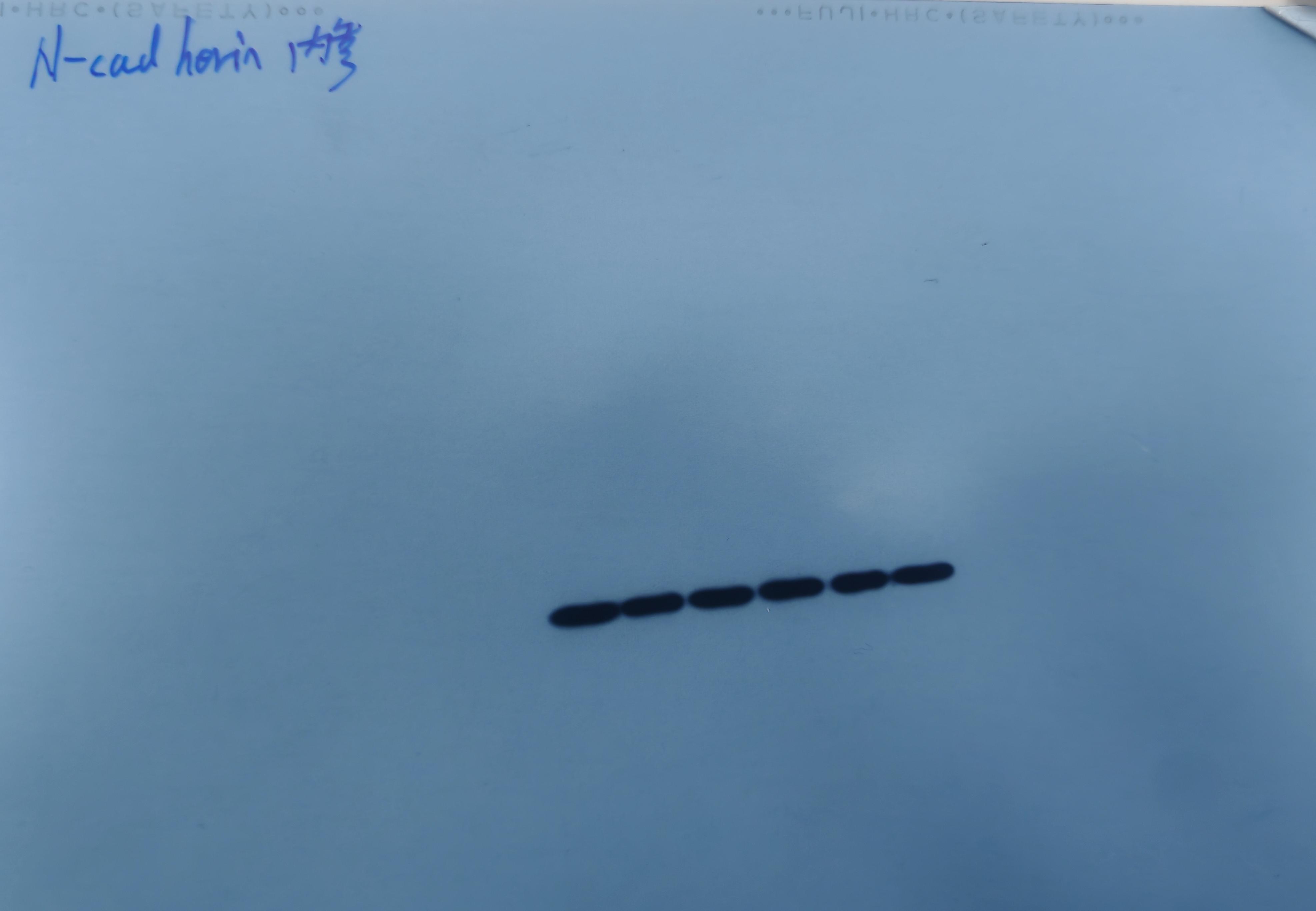

Supplement: Supplementary file 1 [file DataSheet1.zip › Supplymentary/Figure 11. D N-cadherin expression in cells b-actin.jpg]

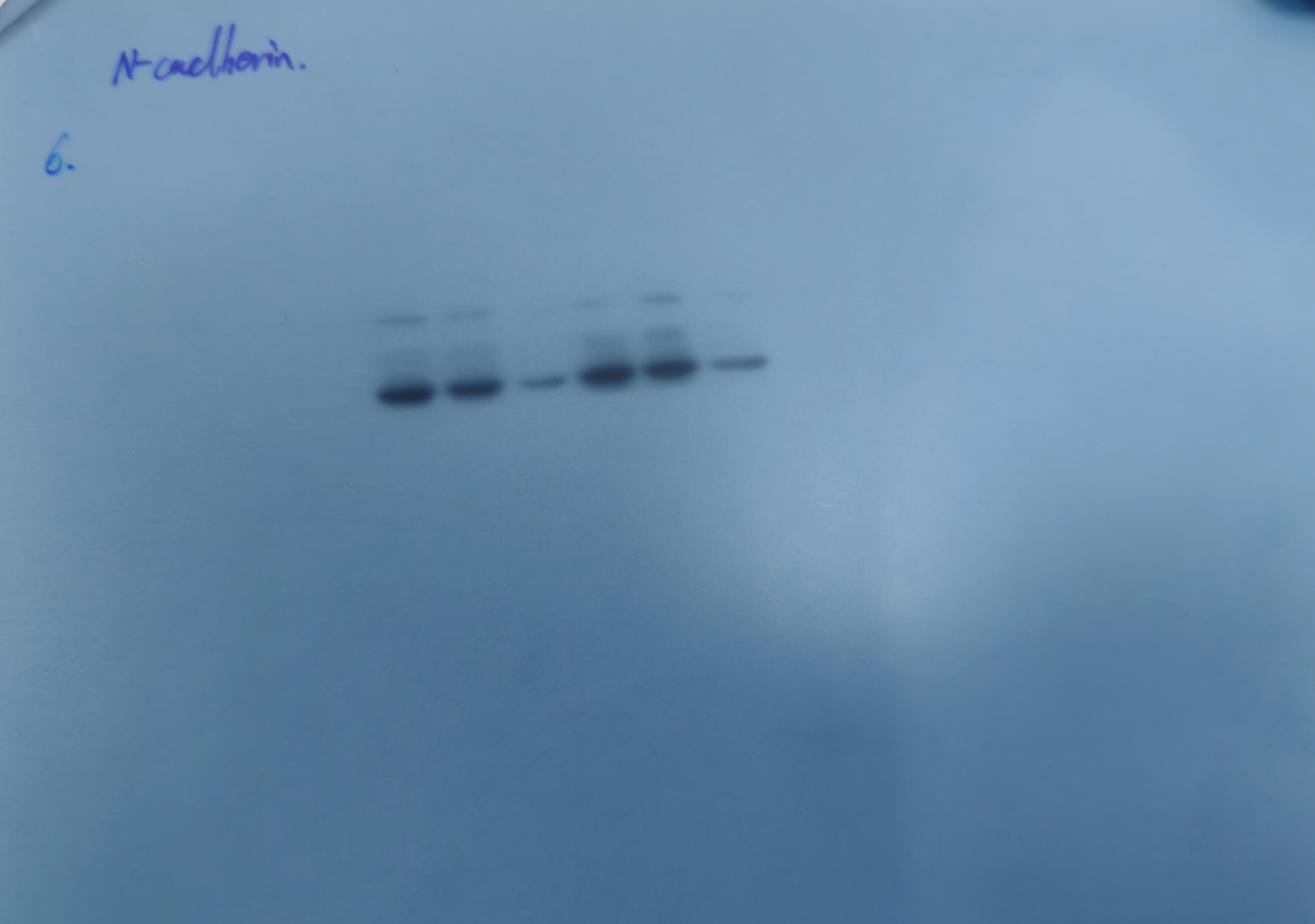

Supplement: Supplementary file 1 [file DataSheet1.zip › Supplymentary/Figure 11. D N-cadherin expression in cells.jpg]

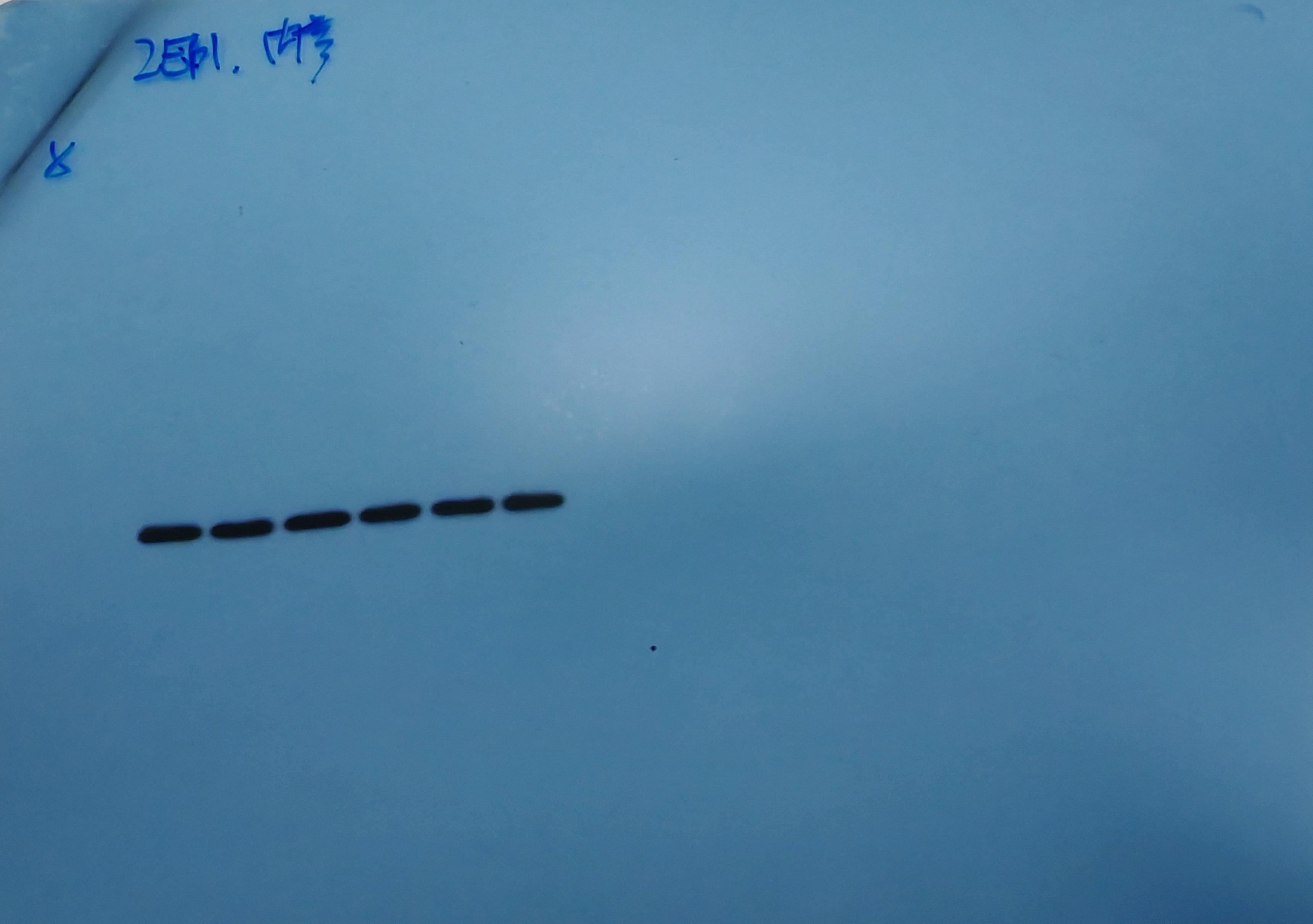

Supplement: Supplementary file 1 [file DataSheet1.zip › Supplymentary/Figure 11. D ZEB-1 expression in cells b-actin.jpg]

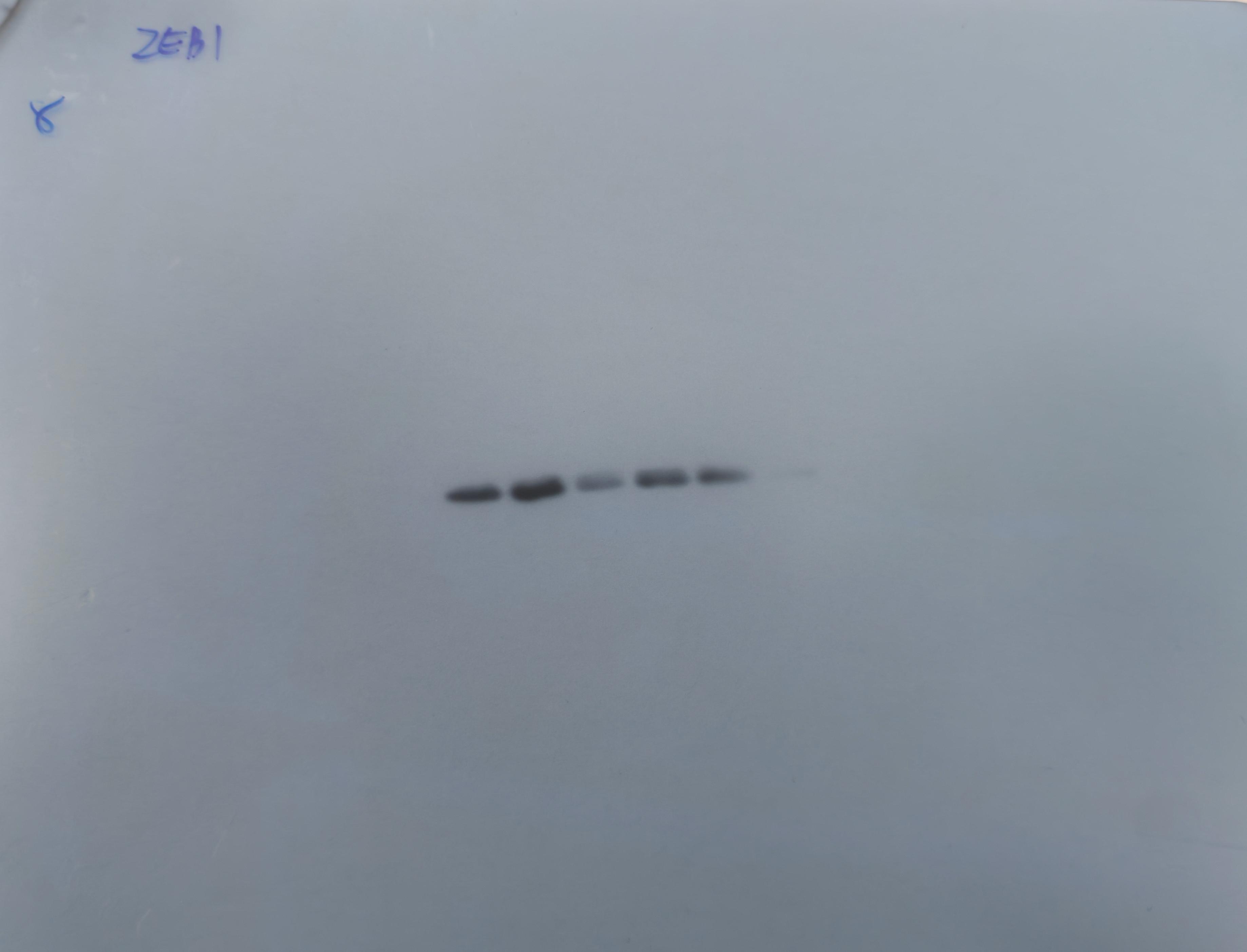

Supplement: Supplementary file 1 [file DataSheet1.zip › Supplymentary/Figure 11. D ZEB-1 expression in cells.jpg]

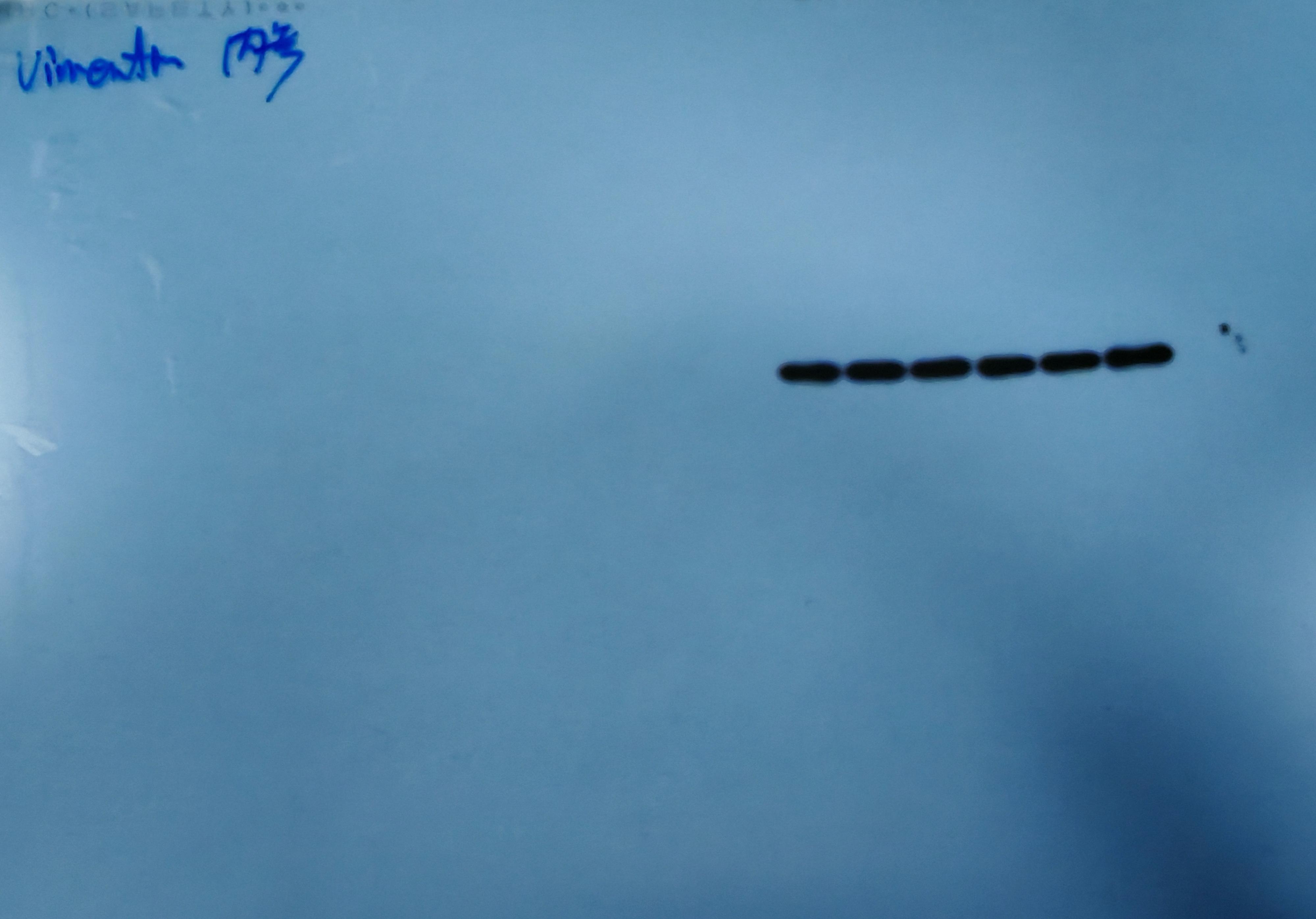

Supplement: Supplementary file 1 [file DataSheet1.zip › Supplymentary/Figure 11. D vimentin expression in cells b-actin.jpg]

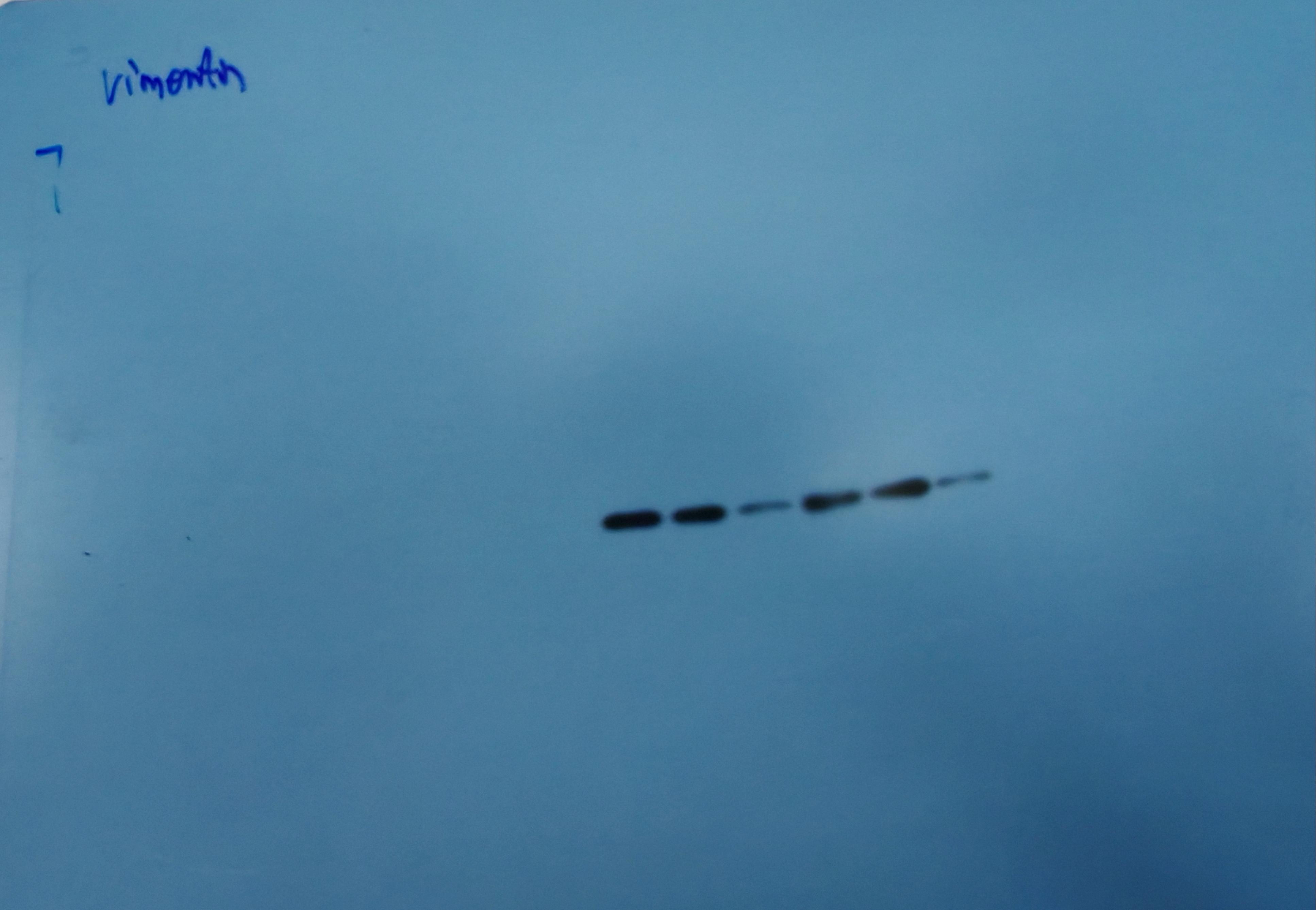

Supplement: Supplementary file 1 [file DataSheet1.zip › Supplymentary/Figure 11. D vimentin expression in cells.jpg]

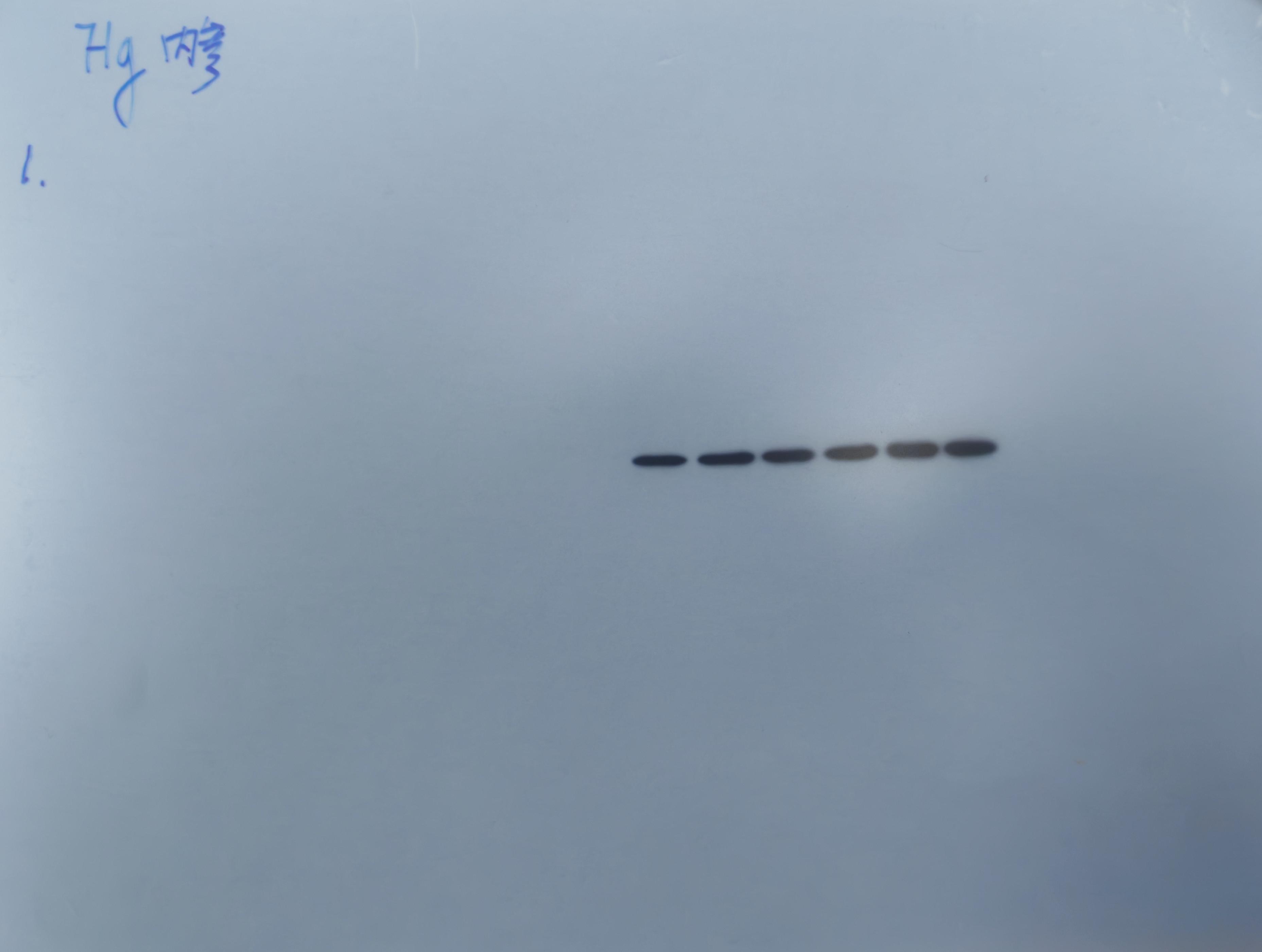

Supplement: Supplementary file 1 [file DataSheet1.zip › Supplymentary/Figure 3. A FLG expression in GC cells b-actin.jpg]

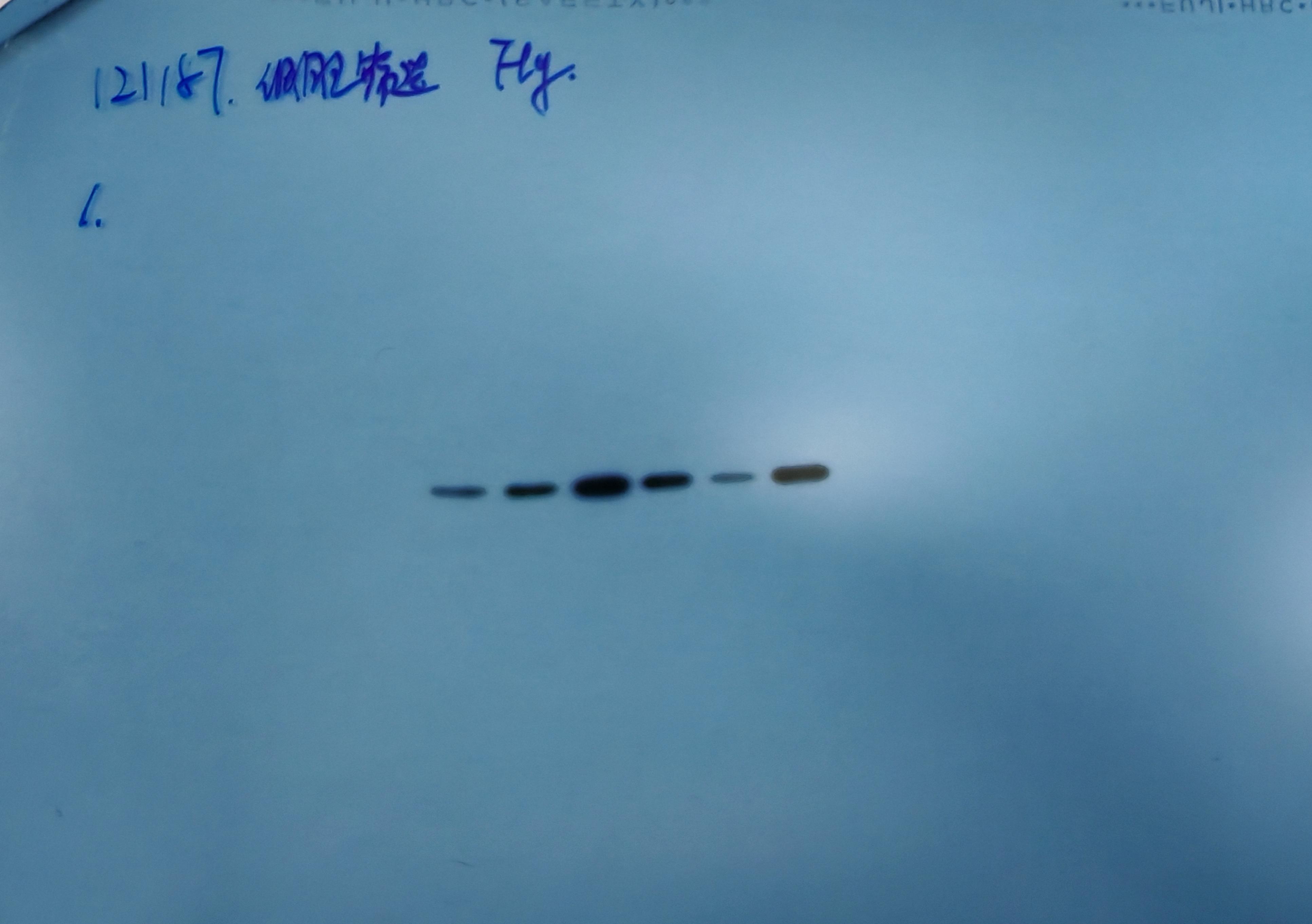

Supplement: Supplementary file 1 [file DataSheet1.zip › Supplymentary/Figure 3. A FLG expression in GC cells.jpg]
